# Effects of Partial and Acute Total Sleep Deprivation on Performance across Cognitive Domains, Individuals and Circadian Phase

**DOI:** 10.1371/journal.pone.0045987

**Published:** 2012-09-24

**Authors:** June C. Lo, John A. Groeger, Nayantara Santhi, Emma L. Arbon, Alpar S. Lazar, Sibah Hasan, Malcolm von Schantz, Simon N. Archer, Derk-Jan Dijk

**Affiliations:** 1 Surrey Sleep Research Centre, Faculty of Health and Medical Sciences, University of Surrey, Guildford, United Kingdom; 2 Department of Psychology, University of Hull, Hull, United Kingdom; 3 Department of Biochemistry and Physiology, Faculty of Health and Medical Sciences, University of Surrey, Guildford, United Kingdom; Vanderbilt University, United States of America

## Abstract

**Background:**

Cognitive performance deteriorates during extended wakefulness and circadian phase misalignment, and some individuals are more affected than others. Whether performance is affected similarly across cognitive domains, or whether cognitive processes involving Executive Functions are more sensitive to sleep and circadian misalignment than Alertness and Sustained Attention, is a matter of debate.

**Methodology/Principal Findings:**

We conducted a 2 × 12-day laboratory protocol to characterize the interaction of repeated partial and acute total sleep deprivation and circadian phase on performance across seven cognitive domains in 36 individuals (18 males; mean ± SD of age = 27.6±4.0 years). The sample was stratified for the rs57875989 polymorphism in *PER3*, which confers cognitive susceptibility to total sleep deprivation. We observed a deterioration of performance during both repeated partial and acute total sleep deprivation. Furthermore, prior partial sleep deprivation led to poorer cognitive performance in a subsequent total sleep deprivation period, but its effect was modulated by circadian phase such that it was virtually absent in the evening wake maintenance zone, and most prominent during early morning hours. A significant effect of *PER3* genotype was observed for Subjective Alertness during partial sleep deprivation and on n-back tasks with a high executive load when assessed in the morning hours during total sleep deprivation after partial sleep loss. Overall, however, Subjective Alertness and Sustained Attention were more affected by both partial and total sleep deprivation than other cognitive domains and tasks including n-back tasks of Working Memory, even when implemented with a high executive load.

**Conclusions/Significance:**

Sleep loss has a primary effect on Sleepiness and Sustained Attention with much smaller effects on challenging Working Memory tasks. These findings have implications for understanding how sleep debt and circadian rhythmicity interact to determine waking performance across cognitive domains and individuals.

## Introduction

How sleep loss and circadian clocks affect brain function is a question with topical relevance because of the negative consequences of inadequate sleep and circadian disruption on health and cognition [1], [2], [3], [4]. Cognitive performance deteriorates during total sleep deprivation (TSD) [5], [6] and the magnitude of this deterioration is considerable such that already after 24 h of wakefulness, performance on a range of measures is as poor as during alcohol intoxication [7]. Cognitive performance also deteriorates during repeated partial sleep deprivation (PSD), and studies have shown that to maintain brain function during the day, young adults require as much as 8–9 h of sleep [8], [9]. Cognitive performance is markedly affected by circadian rhythmicity, independent of sleep. Performance is jeopardized during the circadian night and early morning, even when participants have been awake for less than 16 h [10], [11], but performance is relatively spared in the evening hours during the wake maintenance zone, even when wake duration exceeds 24 h and participants carry a chronic sleep debt [12], [13].

These experiments have established that performance at any given time is determined by an interaction of the duration of the preceding wake episode, the chronic sleep debt carried by the individual, as well as the circadian phase at which performance is assessed. Nevertheless, several issues central to a basic understanding of the modulation of cognitive performance by the sleep-wake cycle and circadian rhythmicity remain unresolved - not least whether the cognitive control processes underpinning the tasks used in these studies are all similarly affected by sleep history and circadian phase. Such underpinning control processes allow us to determine and achieve task goals [14], [15] and include different aspects of Attention (e.g. Sustained, Divided), Memory (e.g. Working, Semantic), as well as three separable components of Executive Functions, i.e. Updating, Task Switching, and Response Inhibition [16].

Tasks that rely on executive processes (e.g. Response Selection and Inhibition) have been reported to be particularly disrupted by sleep deprivation and this has led to the notion that Executive Functions are particularly sensitive to sleep loss [17], [18]. However, a study that investigated the usefulness of various performance tasks to monitor sleepiness-related performance decrements showed that Sustained Attention as assessed by the Psychomotor Vigilance Task (PVT) was more affected during sleep restriction than Response Inhibition as assessed by the Stroop task [19]. In addition, meta-analyses and reviews of TSD studies [3], [20], in which effect sizes of TSD on several cognitive domains were compared across investigations, also suggest that Sustained Attention is as much or more affected than Executive Functions. Furthermore, two recent studies failed to show any particular sensitivity of Executive Functions to the effect of TSD [21], [22]. Thus, the data seem to indicate that the effects of PSD and TSD primarily concern Sustained Attention, with secondary or little effect on Executive Functions. These observations challenge the hierarchical or “cascade” models of cognitive processes, in which Sustained Attention underpins higher-order cognitive functions [20], and concepts of sleep function, in which sleep loss particularly affects cortical neuronal networks involved in executive processes [17], [18].

However, these previous experiments have limitations, including the lack of simultaneous repeated assessment of Sustained Attention and Executive Functions. Multiple assessments of Executive Functions in sleep deprivation studies are rarely undertaken [23], and are challenging to interpret because assessments of Executive Functions typically involve novelty, difficulty, or strategy, all of which are affected by practice. These studies also did not include a direct comparison of the effects of repeated PSD and acute TSD, did not quantify circadian phase, and did not include performance assessment across the circadian cycle. Nor did these previous experiments consider that individuals differ in a trait-like manner in their susceptibility to the effect of sleep deprivation on specific cognitive domains [24], [25]. In none of these studies were study samples prospectively stratified based on genotypic differences, an approach recommended in the study of individual differences [26]. Finally, interpretation of the divergent results related to the differential susceptibility of cognitive domains is hampered because in none of the available studies were all of these variables and factors assessed simultaneously.

We designed an experiment in which we combined repeated PSD and acute TSD and assessments of performance across the entire circadian cycle to investigate whether cognitive domains such as Alertness, Sustained Attention, Working Memory/Executive Functions, as well as Motor and Temporal Control were differentially affected. Executive functions were assessed by using Working Memory tasks (n-back tasks) which maintain the requirement for effective use of core executive processes even after repeated assessment [27]. The study was conducted in a sample of 36 healthy men and women, stratified on the basis of a variable-number (4 or 5) tandem-repeat polymorphism (rs57875989) in the coding region of the clock gene *PERIOD3* (*PER3*) [28] which in previous behavioural [29] and fMRI studies [30], has been identified as a bio-marker for the “trait-like” susceptibility to effects of acute TSD on Working Memory performance with a high executive load when assessed in the morning hours. The data show that cognitive domains are differentially affected by sleep loss such that Alertness and Sustained Attention deteriorate much more than Working Memory, and that these effects are modulated by genotype and circadian phase.

## Results

### Sleep Duration

**Figure 1:**
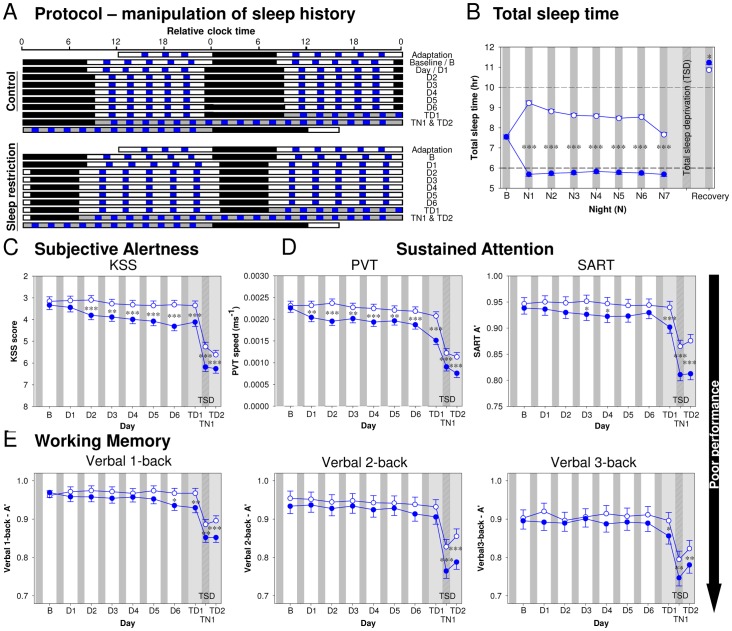
Effect of sleep history on total sleep time and performance. *(* ***A*** *)* Protocol: The study (N = 36) consisted of two 12-day, i.e. 11-night, laboratory sessions as shown in the double raster plot, in which consecutive 24-h periods are plotted both next to and below each other. Following an adaptation night and a baseline night with 8-h Time In Bed (TIB; black bars), sleep opportunity in the subsequent seven nights was either 10 h in the Control condition, or 6 h in the Sleep Restriction (SR) condition. This was followed by a 39-h (Control) or 41-h (SR) acute total sleep deprivation period (grey bars), and a recovery sleep episode (TIB = 12 h). A cognitive performance test battery (blue bars) was administered on the baseline (B) day and each of the following six days (D1 to D6), and during total sleep deprivation (TSD; TD1 = first day of total sleep deprivation; TN1 = night of total sleep deprivation; TD2 = second day of total sleep deprivation). *(* ***B*** *)* Total Sleep Time in the Control (open circles) and SR condition (closed circles). *(* ***C*** *)* Subjective Alertness assessed by the Karolinska Sleepiness Scale (KSS). *(* ***D*** *)* Sustained Attention assessed by the Psychomotor Vigilance Task (speed of the slowest 10% responses) and the Sustained Attention Response Task (A’). *(* ***E*** *)* A’ of Working Memory tasks with increasing executive load (verbal 1- to 2- to 3-back). In all panels, the least square means and standard errors estimated with PROC MIXED in SAS are plotted. Asterisks indicate the significance of the contrast between conditions (***P<0.001, **P<0.01, and *P<0.05).

Thirty-six healthy young men and women (see Table S1 for demographic information) participated in this cross-over design (Figure 1A). Average polysomnographically assessed total sleep time (TST) per 24 h was 8.56±0.06 h in the Control condition and only 5.75±0.06 h in the Sleep Restriction (SR) condition, in which participants were given a 6-h sleep opportunity, i.e. under repeated PSD (SR vs. Control: *F*_1,72.2_ = 2075.68, *P*<0.0001; Figure 1B). During the 12-h recovery sleep episode following acute TSD, TST was significantly longer in the SR than in the Control condition (11.19±0.16 h vs.10.86±0.16 h, *F*_1,28_ = 5.23, *P* = 0.03; Figure 1B). Thus, seven nights of sleep restriction induced a sleep deficit that was carried over the period of acute TSD.

### Effects of Repeated Partial and Acute Total Sleep Deprivation, and Circadian Phase Depend on Cognitive Domain

Participants repeatedly performed a test battery throughout the protocol (Figure 1A; see Materials and Methods for details of the test battery).

Subjective Alertness, i.e. the reverse of the participants’ scores on the Karolinska Sleepiness Scale [31], was stable during the Control condition, but declined steadily during the sleep restriction days (D1 to D6; see Figure 1C). In the subsequent TSD period, Subjective Alertness declined markedly from the first day (TD1), through the following biological night (TN1), with a limited recovery the next day (TD2).

Sustained Attention was assessed with the Psychomotor Vigilance Task (PVT) [32] in which participants wait an unpredictable amount of time for a single discrete stimulus before making a simple, well-practised response. Response speed of the 10% slowest responses deteriorated during PSD and TSD (Figure 1D; see Figure S1A and Supporting Information for the results of lapses). Sustained Attention was further evaluated with the Sustained Attention to Response Task (SART) [33] in which a simple, well-practised response is made to the vast majority of rapidly presented stimuli, and withheld when a target stimulus is presented. Performance on the SART was quantified taking both errors of omission and commission into account by computing an accuracy measure, A’ (refer to the statistical methods section for details), which deteriorated during PSD and TSD (Figure 1D).

We assessed Working Memory and Executive Functions with verbal n-back tasks in which participants repeatedly compare the current stimulus with the one presented 1 to 3 items before. As task difficulty increases from 1- to 3-back, so does the load on the three components of Executive Functions, i.e. updating of maintained information, task switching (e.g. between updating and memory comparison), and response selection [16]. Performance which was also quantified by A’ varied significantly across the three n-back tasks with poorest performance observed on the 3-back (main effect of Level on A’: *F*_2,68_ = 26.71, *P*<0.0001 and for each pair-wise comparison, *P*<0.0001; on a measure of response tendency, bias [B”_D_]: *F*_2,68_ = 29.92, *P*<0.0001 and for each pair-wise comparison, *P*<0.05). Although the negative effect of TSD on n-back performance was substantial, especially during TN1, and a smaller effect of PSD was observed, n-back tasks with a higher executive load did not appear to be more affected by sleep deprivation than those with a lower executive load (Figure 1E). On the other hand, B”_D_ was more sensitive to the effects of PSD. After two or four nights of sleep restriction, participants became more conservative in their responses, i.e. increased their tendency to claim that ‘matches’ were not occurring (see Figure S1B).

For the statistical evaluation of these effects and for the comparison of the size of the effects across tasks, we first analysed all segments of the protocol combined (i.e. baseline, D1–D6, TD1, TN1, and TD2; see Table S2 for statistical results). We computed the implied effect size (*f^2^*; refer to the statistical methods section for details) and found that the effect of Condition, i.e. Sleep Restriction vs. Control, was greater for Subjective Alertness and Sustained Attention measures than for Working Memory (Figure S2). For the latter tasks, the effects of Condition were small and independent of the level of executive demand (Condition x Level interaction on A′: *F*_2,68_ = 0.38, *P* = 0.68; B″_D_: *F*_2,68_ = 0.47, *P* = 0.63).

**Figure 2:**
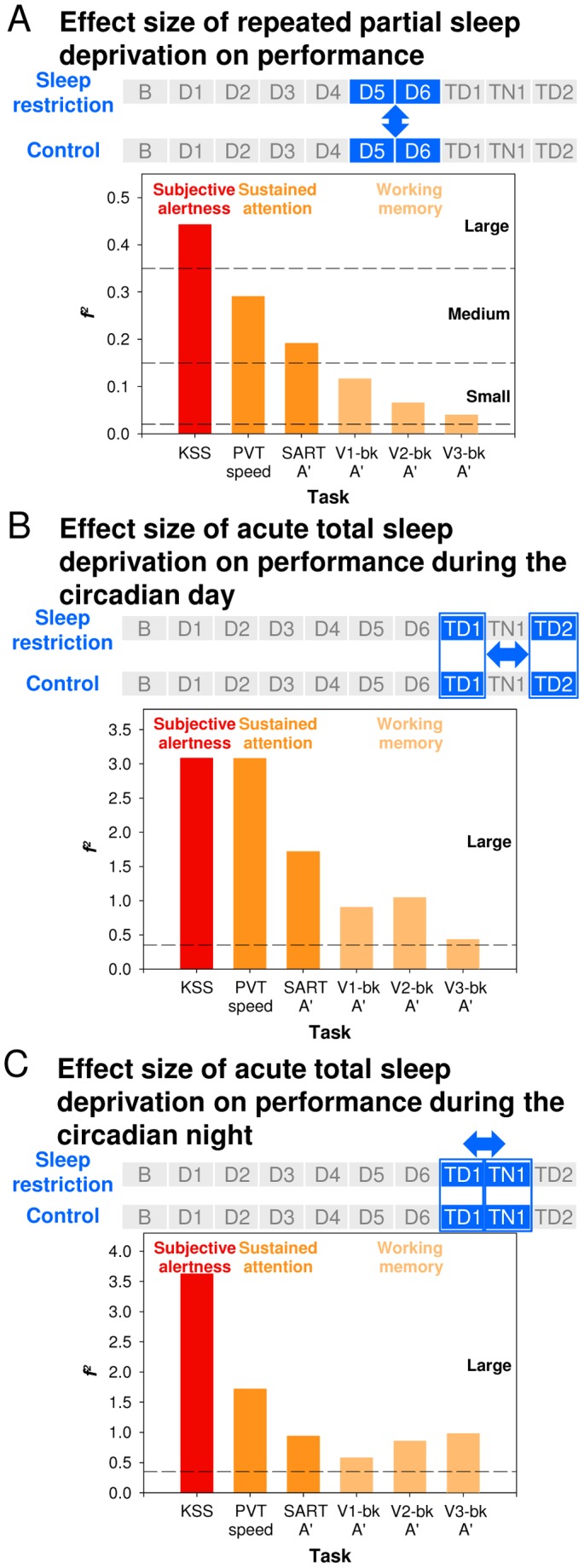
Comparison of effect sizes for Subjective Alertness, Sustained Attention, and Working Memory. *(* ***A*** *)* Effect size of repeated partial sleep deprivation. It was assessed by comparing performance during D5 and D6 between conditions. *(* ***B*** *)* Effect size of acute total sleep deprivation on performance during the circadian day. It was assessed by comparing performance on TD1 to performance on TD2 across conditions. *(* ***C*** *)* Effect size of acute total sleep deprivation on performance during the circadian night. It was assessed by comparing performance on TD1 to performance in TN1 across conditions. Horizontal lines indicate cut-offs for small, medium, and large effect sizes.

We next determined whether repeated PSD and acute TSD had differential effects on performance by contrasting effects in different segments of the protocol. The effect of **repeated PSD** was estimated by comparing performance after five and six nights (D5 and D6) of restricted sleep opportunities to performance during D5 and D6 in the Control condition. We found a significant effect of repeated PSD on Subjective Alertness, Sustained Attention, and all measures of Working Memory performance except for B″_D_ in the verbal 3-back task (Table S3). The effect size of repeated PSD varied considerably across the various cognitive domains. By conventional metrics [34], the effect size was large for Subjective Alertness, medium for the Sustained Attention measures, and small for Working Memory (Figure 2A). For the Working Memory tasks, the effect of repeated PSD was independent of the level of executive load (Level × Condition interaction on A′: *F*_2,67_ = 0.14, *P* = 0.87; B″_D_: *F*_2,67_ = 0.58, *P* = 0.56).

When we contrasted all the 52 performance measures of the seven cognitive domains between the Control and the SR conditions, we found a near linear increase in the effect of repeated PSD from D1 to D6 (Figure S3).

To further examine the differential effects of repeated PSD on Sustained Attention and Executive Functions, we contrasted the change of performance at the end of the sleep history manipulation period relative to the baseline day (i.e. D6/baseline) in the SR and the Control conditions. After six nights of restricted sleep opportunities, PVT performance was at a 79.86±1.04% (mean speed of the slowest 10% responses ± SEM) level relative to baseline, while at the end of the Control period, performance was at a 93.38±1.04% level, and these changes in performance across the sleep history manipulation period differed between the two conditions (*F*_1,33.8_ = 11.49, *p* = 0.002). In contrast, verbal 3-back performance (A′) at the end of the sleep history manipulation period was at a 99.36±1.01% level in the SR condition and 100.15±1.01% level in the Control condition relative to baseline, and no significant main effect of Condition was found (*F*_1,34.1_ = 0.19, *p* = 0.66). These results emphasized that repeated PSD impaired Sustained Attention, while its influence on Executive Functions was minimal.

The effect of **acute TSD** was estimated by comparing performance on TD1 to performance on TD2 of the TSD segment. These performance measures were obtained at the same circadian phase and only differed with respect to the duration of the wake episode preceding the performance assessments. TSD impaired all performance measures (Table S3), but the detrimental effects were more prominent for Subjective Alertness and Sustained Attention measures than for Working Memory (Figure 2B). The effect of TSD on Working Memory was independent of the level of executive load (Level × Condition interaction on A′: *F*_2,175_ = 1.12, *P* = 0.33; B″_D_: *F*_2,175_ = 2.95, *P* = 0.05).

To further investigate the differential effects of acute TSD on Sustained Attention and Executive Functions, we contrasted the change of performance on the second relative to the first day of the TSD period (i.e. TD2/TD1) in the SR and the Control conditions. Relative to the first day of TSD, PVT speed on the second day was reduced by about 50% in both the SR and the Control conditions (mean ± SEM: 46.28±1.08% vs. 49.46±1.08%, *F*_1,32.7_ = 0.48, *p* = 0.49), while verbal 3-back performance was maintained at an approximately 90% level (A’ in SR vs. Control: 90.64±1.03% vs. 91.81±1.03%, *F*_1,34.1_ = 0.14, *p* = 0.71). These findings further indicated the greater detrimental effects of acute TSD on Sustained Attention than on Executive Functions.

We also evaluated the effects of TSD on performance during the **circadian night** by contrasting performance on TD1 and TN1. As in the previous comparisons, the effects of TSD were larger on Subjective Alertness and Sustained Attention (particularly reflected in PVT speed) than on Working Memory performance (Figure 2C).

This indicates the robustness of these differential effects of sleep deprivation on various cognitive domains.

The effects of repeated PSD and acute TSD were computed for other measures of these tasks, e.g. lapses of attention and speed on the 10% fastest response in the PVT, errors of omission and commission of the SART, as well as tasks including measures of Motor and Temporal Control and subjective assessments of Workload (see Figure S4A and B). Even when all these measures were considered, Sustained Attention and subjective measures, including those of Workload, remained most affected by the sleep manipulations and particularly so by acute TSD.

### PER3 Polymorphism Modulates Effects of Partial and Total Sleep Deprivation on Alertness and Working Memory with a High Executive Load

The three *PER3* genotypes obtained similar amounts of sleep (main effect of Genotype on TST: *F*_2,33.9_ = 0.89, P = 0.42) and their TST was not differentially affected by the two conditions (Condition × Genotype: *F*_2,76.1_ = 0.74, *P* = 0.48).

**Figure 3:**
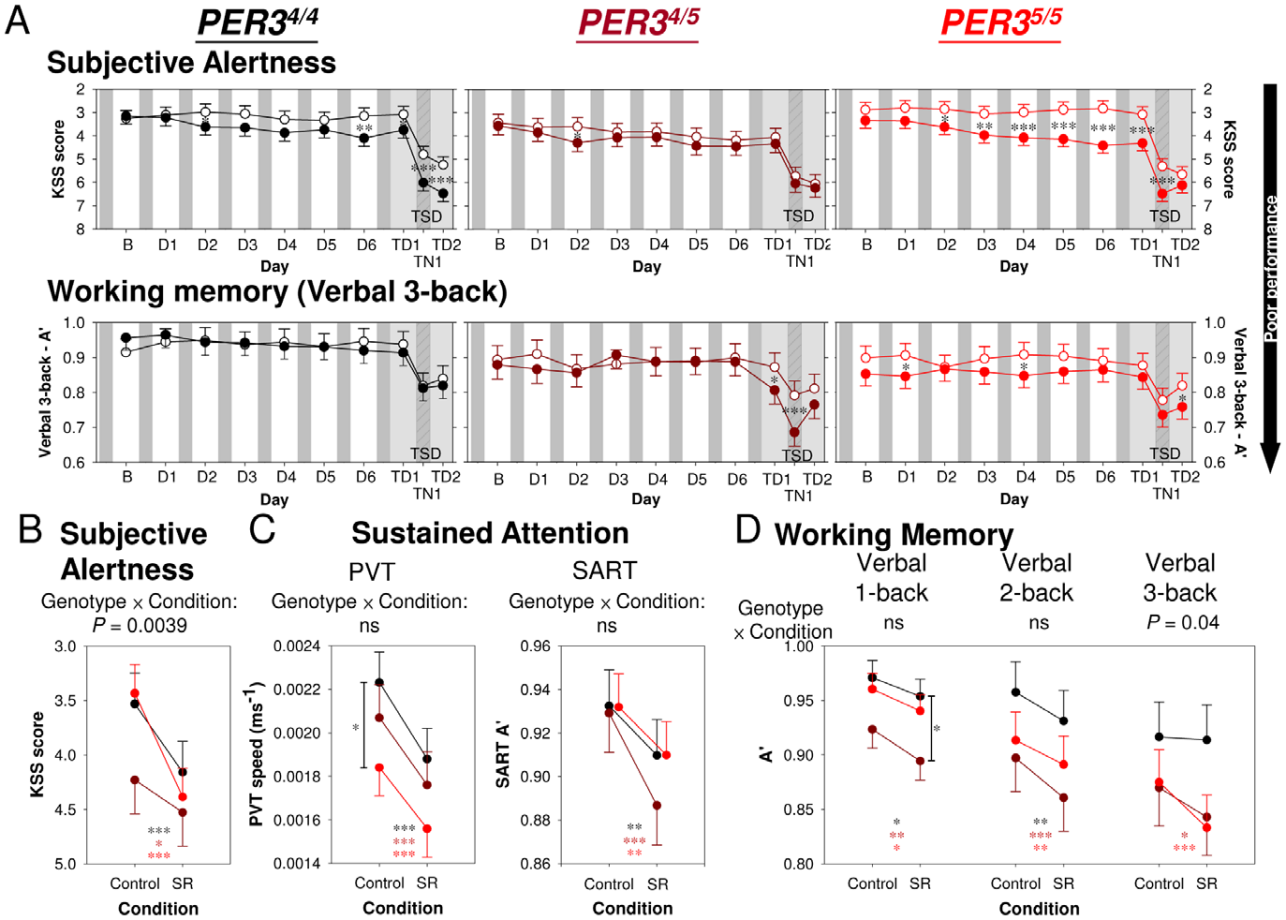
Effect of *PER3* genotype on performance during repeated partial and subsequent acute total sleep deprivation. *(* ***A*** *)* Time course of Subjective Alertness (top panel) and Working Memory/Executive Functions (bottom panel) in *PER3^4/4^*, *PER3^4/5^*, and *PER3^5/5^* individuals. The least square means and standard errors estimated with PROC MIXED in SAS are plotted. Asterisks indicate the significance of the contrast between conditions (***P<0.001, **P<0.01, and *P<0.05). Open circles = Control condition; filled circles = Sleep Restriction (SR) condition. *(* ***B, C, D*** *)* Performance during the SR and the Control conditions averaged throughout the protocol (B - TD2) in the three genotypes. The interaction between Genotype and Condition was significant for *(* ***B*** *)* Subjective Alertness (*P* = 0.0039) and for *(* ***D*** *)* Working Memory**,** but only for verbal 3-back (*P* = 0.04). For neither of the *(* ***C*** *)* Sustained Attention measures was the Genotype × Condition interaction statistically significant (ns = not significant).

The time course for selected performance measures throughout the protocols was assessed separately for the three genotypes. When the entire protocol was considered, there was no significant effect of Genotype on Subjective Alertness, but the interaction between Genotype and Condition was significant (Figure 3A and B; Table S4) such that the negative effect of sleep restriction on Subjective Alertness was greatest in the *PER3^5/5^* participants. The effect of Genotype or Genotype × Condition interaction was not significant for the Sustained Attention measures (Figure 3C; see Figure S5A for the time course and Table S4 for the statistics). When all segments of the protocol were considered, no significant effect of Genotype was observed for any of the Working Memory measures, and the interaction between Genotype and Condition was significant in just one circumstance (Table S4). Only for A’ in the verbal n-back task with the highest executive load, i.e. verbal 3-back, did we observe a significant interaction (*F*_2,155_ = 3.42, *P* = 0.04; Figure 3A and D), indicating that sleep restriction affected 3-back accuracy differently across the *PER3* genotypes. Specifically, the performance of the *PER3^4/4^* homozygotes was not affected by sleep history during either the sleep restriction days or subsequent TSD, while in the *PER3^4/5^* heterozygotes, negative effects of sleep restriction were observed during the TSD period only. In the *PER3^5/5^* homozygotes, a decrement in verbal 3-back performance was quite consistently found after the first night of 6-h sleep opportunity, and was also observed during subsequent TSD (see Figure S5B for a similar time course of B”_D_). These differential responses of the *PER3* genotypes to sleep loss were not found in Working Memory tasks with a smaller executive load, i.e. 2-back, or in relatively undemanding Working Memory, i.e. 1-back (Figure 3D).

Comparison of the effect size of the interaction between Genotype and Condition across these tasks indicates that when all segments of the protocol are considered, Subjective Alertness and verbal 3-back performance during sleep loss were most influenced by Genotype (Figure S6). The *PER3* genotypes did not differ in their responses to sleep deprivation for Sustained Attention or Working Memory tasks with small load on Executive Functions.

**Figure 4:**
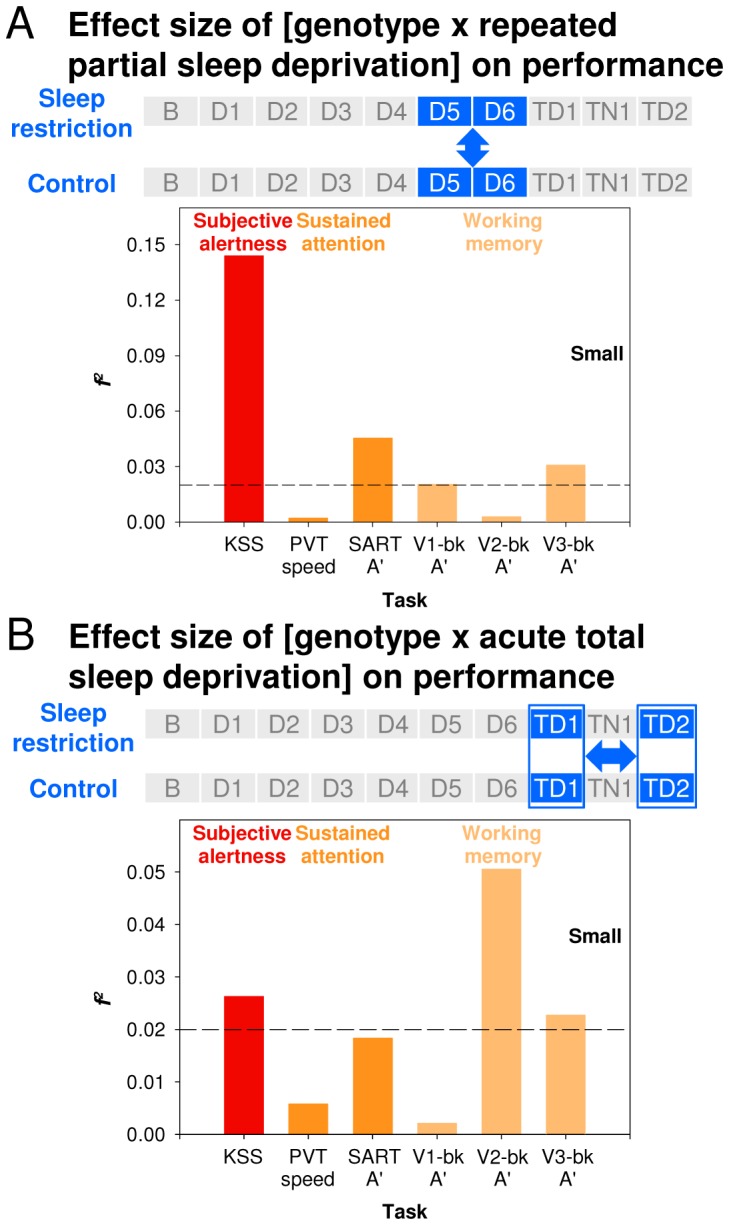
Comparison of the effect sizes for the Genotype × Sleep Deprivation interaction across cognitive domains. *(* ***A*** *)* During partial sleep deprivation. It was assessed by comparing performance during D5 and D6 between conditions. *(* ***B*** *)* During total sleep deprivation. It was assessed by comparing performance on TD1 to performance on TD2 across conditions. Horizontal lines indicate cut-offs for small effect sizes.

Analyses conducted separately for the effects of repeated PSD and acute TSD revealed that whereas the Genotype effect on Subjective Alertness was more pronounced for PSD (Figure 4A), the Genotype effect on Executive Functions was almost exclusively related to the TSD effect (Figure 4B). Thus, the *f^2^* of the Genotype × PSD interaction on KSS score was 5.5 times larger than that of the Genotype × TSD interaction. The *f^2^* of the Genotype × TSD interaction on verbal 2-back performance was 18.2 times larger than that of the Genotype × PSD interaction. For speed measures on the PVT, effect sizes of Genotype were relatively small for both PSD and TSD. Thus, largest effects of *PER3* genotype during acute TSD concern different cognitive domains than during repeated PSD.

Please note that even in the *PER3^5/5^* participants, the effect of TSD was greater on Sustained Attention (the speed of the slowest 10% responses in the PVT) than on Working Memory (Figure S7).

### Circadian Rhythmicity Modulates Effects of Partial and Total Sleep Deprivation on Cognitive Performance

Circadian rhythmicity itself was affected by PSD. After repeated PSD, the circadian rhythm of melatonin was delayed by 45 minutes (mean ± standard error of dim light melatonin onset [DLMO]: 00∶13±00∶15 vs. 23∶28±00∶15; main effect of Condition: *F*_1,30_ = 28.15, *P*<0.0001; Figure S8; see Table S5 for the effects of Condition and Genotype on other circadian phase markers). For all subsequent analyses of the effect of Circadian Phase, the behavioural data during TSD were aligned to the melatonin rhythm.

**Figure 5:**
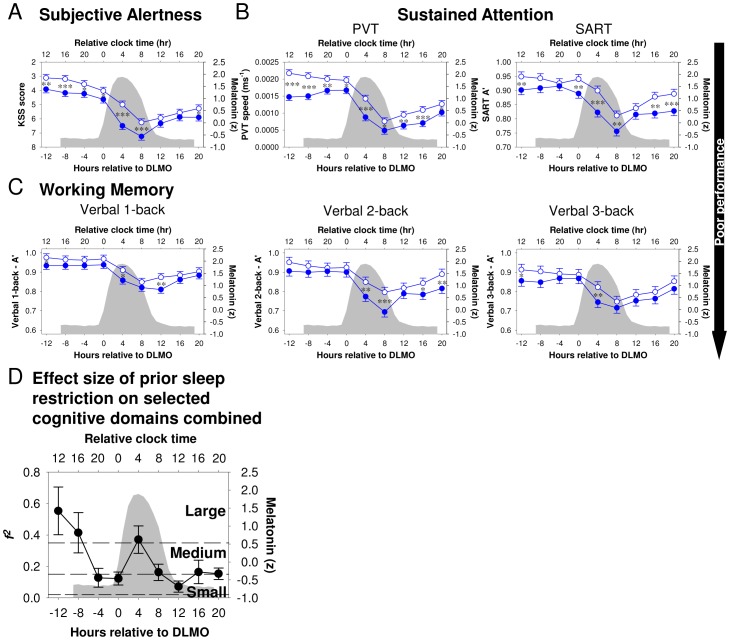
Effects of sleep history on circadian modulation of performance during total sleep deprivation. Time course of *(* ***A*** *)* Subjective Alertness, *(* ***B*** *)* Sustained Attention, and *(* ***C*** *)* Working Memory during total sleep deprivation (TSD) in the Sleep Restriction (filled circles) or the Control condition (open circles) across different circadian phases relative to dim light melatonin onset (DLMO; grey filled area = the melatonin profile averaged between the two conditions). *(* ***D*** *)* Effects size of prior partial sleep deprivation on performance during TSD per 4-h circadian melatonin bins averaged across the six performance measures. Error bars represent the between performance measure standard error of the mean. Horizontal lines indicate cut-offs for small, medium, and large effect sizes.

Prominent circadian variation was observed for Subjective Alertness, Sustained Attention, and Working Memory during the TSD period (Table S6). For all of these measures, the minimum was observed in the morning hours, i.e. DLMO +8 h (Figure 5A–C). Following repeated PSD, all performance measures were lower throughout the TSD period (Table S6 and Figure 5A–C). However, the effect of repeated PSD on the time course of performance during TSD was not constant (Figure 5A–C). For most of the measures, the effect was greatest in the initial part of the TSD period, then became smaller, with a minimum in the evening hours close to the DLMO, and increased again during the circadian night with a maximum at around DLMO+4 h. On the second day of TSD, effect of prior PSD was, in most cases, smaller (Figure S9). Indeed, we found a significant Condition × Circadian Phase interaction for Subjective Alertness and PVT measures, though not for SART or n-back measures (Table S6).

For the Working Memory performance measures, pairwise comparisons of performance per circadian bin suggest that significant effects of prior PSD were mainly observed in the initial part of the TSD period and during the biological night at DLMO +4 h (Figure 5C).

Averaging the effects sizes of prior PSD across these cognitive domains but separately per circadian phase bin revealed the general pattern of largest effects in the morning and afternoon during the initial hours of TSD, followed by a decline of the effects with a minimum in the evening hours at around the DLMO, followed by a sudden subsequent increase during the biological night (Figure 5D; effect of Circadian Phase: *F*_8,40_ = 6.33, *P*<0.0001). This circadian modulation of the effects of sleep history on performance decrements during TSD was confirmed by analysing the effect sizes of all measures of the performance battery including Temporal and Motor Control and subjective assessments of Workload (see Figure S10A; effect of Circadian Phase: *F*_8,408_ = 28.96, *P*<0.0001).

### PER3 Genotype Modulates Circadian Rhythmicity Effects on Cognitive Performance

**Figure 6:**
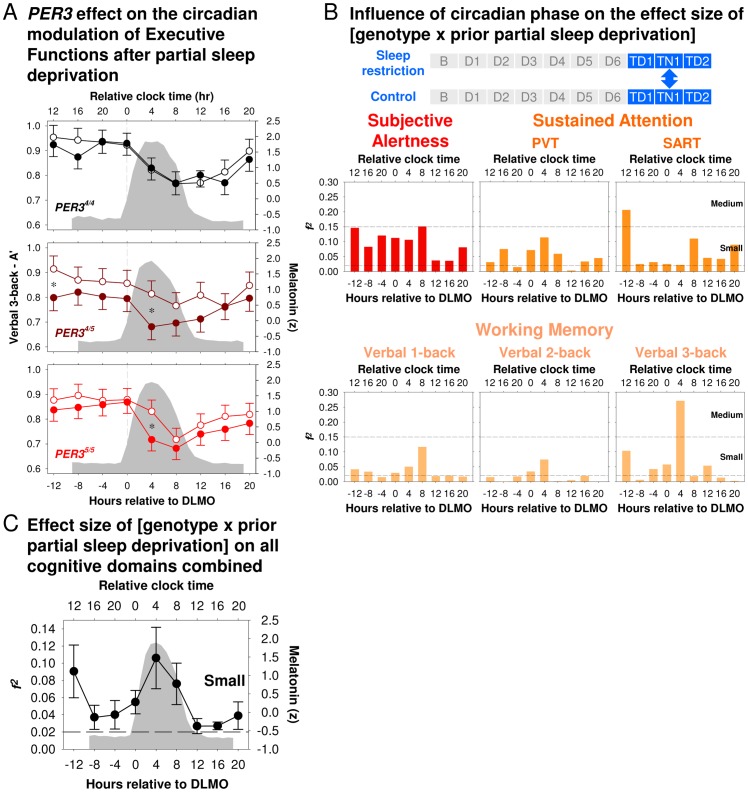
Effects of *PER3* genotypes on the circadian modulation of performance during total sleep deprivation following partial sleep deprivation. *(* ***A*** *)* Verbal 3-back performance during total sleep deprivation (TSD) in the Sleep Restriction (filled symbols) and the Control conditions (open circles) separately for the three *PER3* genotypes. (DLMO: dashed grey vertical line and melatonin profile averaged between the two conditions shaded in grey). *(* ***B*** *)* Effect sizes for the Genotype × Sleep History Condition interaction during TSD for Subjective Alertness, Sustained Attention, and Working Memory, computed for each 4-h circadian melatonin bin. *(* ***C*** *)* Effect size averaged across the six performance measures. Error bars represent the between performance measure standard error of the mean. Horizontal lines indicate cut-offs for small and medium effect sizes.

*PER3* genotype modulated the interaction between Circadian Rhythmicity and Sleep History but to a different extent for Executive Functions than Subjective Alertness, Sustained Attention, and Working Memory with a low executive load. In the *PER3^4/4^* homozygotes, verbal 3-back performance during sustained wakefulness was not affected by prior PSD at any circadian phase. By contrast, in the *PER3^4/5^* heterozygotes and the *PER3^5/5^* homozygotes, verbal 3-back performance was poorer following PSD especially at DLMO+4 h, which on average corresponded to 02∶00 to 06∶00 (Figure 6A). This circadian modulation of the effect of Genotype × Sleep History interaction was confirmed by comparing the effect size of the Genotype × Condition interaction on performance during TSD at various circadian phases for Subjective Alertness, Sustained Attention, and the verbal n-back tasks. Although for most of the performance measures, effect sizes were in general larger at the beginning of the TSD period during the circadian night, and in the post-TSD morning than in the post-TSD afternoon, the largest *f^2^* was observed for the verbal 3-back performance at DLMO+4 h, i.e. between 02∶00 and 06∶00 (Figure 6B). A general pattern of largest effects of Genotype in the morning during the initial hours of the TSD, followed by a decline of the effects throughout the biological day and a sudden subsequent increase during the biological night was revealed by averaging the effects sizes of Genotype across these cognitive domains but separately per circadian phase bin (Figure 6C; effect of Circadian Phase: *F*_8,40_ = 2.43, *P* = 0.03). The standard errors of the effect sizes co-varied with the average effect size, indicating that not only the effect sizes vary with circadian phase but also the extent to which different cognitive domains are differentially affected. This was further confirmed by analysing the effect sizes of all measures of the performance battery (see Figure S10B; effect of Circadian Phase: *F*_8,408_ = 2.65, *P*<0.01), such that largest effects of Genotype and largest divergence between tasks were observed in the morning hours, with smaller effects observed in the evening and afternoon.

## Discussion

The protocol allowed for a comprehensive assessment of the interaction of circadian rhythmicity, chronic partial and acute total sleep loss, and *PER3* genotype on performance across cognitive domains under carefully controlled laboratory conditions in a substantial sample of healthy young adults. The data show that Subjective Alertness and Sustained Attention are more affected by both repeated PSD and acute TSD than Executive Functions, as assessed by a Working Memory task implemented with a high executive load. Furthermore, circadian rhythmicity modulates the effects of sleep history such that the brain appears not much affected by sleep loss when performance is assessed during the wake maintenance zone in the evening hours, whereas in the morning hours, brain function is much more vulnerable. In addition, individual differences in the susceptibility to the effects of sleep loss, as predicted by *PER3* genotype, primarily concern Subjective Sleepiness during PSD and Executive Functions (e.g. Updating, Task Switching) during TSD, rather than Sustained Attention. Differences between genotype and tasks in response to sleep loss are, however, dependent on the circadian phase at which performance is assessed. Together, these findings challenge the notion that Executive Functions/Working Memory are particularly susceptible to the negative effects of sleep loss [17], [18]. Instead, they imply that sleep-wake history and circadian phase primarily modulate Sustained Attention and that Executive Functions are not necessarily affected when Sustained Attention is compromised. Greatest divergence between genotypes and variation across tasks were observed in the morning hours after TSD. These findings have practical and theoretical implications.

### Validity of Protocol and Performance Measures

The longer TST during recovery sleep after Sleep Restriction relative to the Control conditions (Figure 1B) suggests that limiting the sleep opportunity to 6 h per 24 h was successful in creating a chronic sleep debt that was carried over the period of TSD. The relatively small difference in TST (approximately 20 min) during the recovery sleep episodes probably reflects a ceiling effect since TST was close to 11 hours during the 12 h sleep opportunity. In the Control condition, during which performance did not deteriorate, participants on average slept 8.56 h. This is close to a previous estimate of the sleep duration required to maintain performance [8], [9] and only slightly less than the 8.9 h which is the estimated maximal capacity for sleep in young adults when given 16 h of sleep opportunity per 24 h [35].

The data reported above provide several indications for the validity of our performance measures. The magnitude of changes in Subjective Alertness observed in the current study, are comparable to previous reports [8], [9]. Our measures of Sustained Attention behaved as expected: the speed of the 10% slowest responses on the PVT was much more affected by repeated PSD and acute TSD than the 10% fastest responses. This is in accordance with previous behavioural studies [36], [37] and provides evidence for differential brain correlates of the slowest and fastest responses as assessed in fMRI studies [38], [39]. Consistent with previous reports on the effects of sleep deprivation on the SART, which involves Sustained Attention and Response Inhibition [40], both errors of omission and commission were affected by sleep deprivation, but in our data, the accuracy measure, A’, was shown to be more sensitive than either of the error measures (Figure S4). Performance on the Working Memory tasks with varying executive load displayed characteristics in accordance with the intended targeted processes. The progressive reduction of performance from 1- to 2- to 3-backs is consistent with existing findings [27] and was observed throughout the protocol. Moreover, as A’ was above chance level, we are confident that the observed decrease in accuracy and increase in tendency towards more conservative responses were associated with task difficulty and that participants continued to engage genuinely with the tasks even after repeated administration.

### The psychological Properties of the Cognitive Tasks Used

In the present study, we compared the effects of sleep deprivation across tasks carefully selected to recruit different cognitive domains. These tasks have been widely used in cognitive research, in both laboratory and neuroimaging studies, as well as in sleep research. We used multiple tasks, with known redundancies and partial functional overlaps (e.g. 1-back verbal, spatial, visual, integrated spatial and visual) in order to counter the inherent difficulty with cognitive tasks that no task is ‘process pure’. That is, even apparently simple tasks may depend upon multiple simple processes. These simpler processes are themselves more or less influential in performance as tasks are, wittingly or otherwise, performed differently by participants on different occasions (e.g. context, time-pressure, practice, speed-accuracy trade-offs, feedback, reward, etc.). By using multiple tasks that had been extensively practiced, and almost all of which are externally paced, with minimal direct knowledge of results, we believe we can minimise many of these unwanted sources of performance variability. Our multi-task approach provides an alternative to other approaches in which the effects of sleep deprivation are investigated on the cognitive components of a single task, the interaction between which is prey to all of the influences identified above [22].

### Effect Sizes of Repeated Partial and Acute Total Sleep Deprivation across Cognitive Domains

Computation of effect sizes [34], [41] and the simultaneous and repeated assessment of performance in many cognitive domains allowed for a unequivocal demonstration that the effects of **repeated PSD** and **acute TSD** on Subjective Alertness and Sustained Attention are larger than effects on Working Memory/Executive Functions. In fact, little evidence for a particular sensitivity of the executive component of the n-back tasks to the effects of sleep loss was observed in the present study. Importantly, no interaction between executive load and either PSD or TSD was observed for A′ or B″_D_. The fact that throughout the present study, performance was reliably affected by task difficulty, allows us to be more conclusive regarding the lack of a relationship between task difficulty and the negative effects of sleep deprivation, since in previous studies, simpler and more difficult tasks were impaired by sleep deprivation to similar extents [42], [43], [44].

Meta-analyses of studies on the effects of acute TSD on accuracy and speed measures of performance across a wide range of cognitive domains indicated that Sustained Attention was particularly affected, although only few studies included in the meta-analyses used n-back tasks [20] and none assessed the effect of repeated PSD on Executive Functions as assessed by the n-back tasks. In our study, effects of sleep deprivation on Sustained Attention were not only greater than effects on Working Memory, but also greater than effects on measures of Temporal and Motor Control (Figure S4). The differential sensitivity of performance across cognitive domains was observed during the circadian night and day and following repeated PSD and acute TSD. In addition, in the current data set, the effects of TSD were larger than the effects of PSD, but the relative sensitivity of the various tasks appeared similar for the two interventions.

We used effects sizes, which are widely used in the psychological literature and also in meta-analyses, as our primary tool to compare the effects of sleep deprivation across tasks. The validity of this choice was confirmed by computing relative changes in performance for some of the tasks and these measures also showed that Sustained Attention task was more affected than Working Memory task even when implemented with a high executive load. In the current protocol, we also directly compared the effects of PSD and TSD and across many different tasks, thereby providing yardsticks both within and across tasks. Effect sizes, although useful in comparing across tasks, interventions, and studies, do, of course, not inform about the ‘clinical’ significance of the effect. This would require comparing the effects of sleep loss on tasks to the effects observed in specific patient populations, or using tasks that have in a quantitative way been correlated with performance in the real world.

### Interaction Effects of Circadian Phase and Sleep Homeostasis on Cognition

In our protocol, sleep restriction was accomplished by a symmetrical change in bedtime and wake time because we wanted to keep the centre of light exposure unchanged and thereby minimise circadian phase shifts. Nevertheless, repeated PSD led to a delay of the melatonin rhythm. This finding provides further evidence [45] for a feedback of the sleep homeostat onto the circadian timing system and emphasizes the necessity to assess circadian phase. In contrast to previous studies [22], [46], performance data were aligned with the melatonin rhythm, which is particularly important when group averages are computed across individuals with different circadian phases. These analyses demonstrated that the effects of sleep history on cognition were modulated by circadian phase. During acute TSD, all cognitive performance measures displayed a near stable performance during the first day, a steep decline during the biological night, and some recovery on the second day [10], [47], suggesting that one circadian signal modulates all these performance measures.

The effects of sleep history were greatest in the initial part of the acute TSD, became progressively smaller and then increased again during the biological night. In our protocol, sleep restriction was accomplished by a symmetrical change in bed time and wake time; hence, compared to the Control condition, in the SR condition, wake time occurred at an earlier clock time (2 h) and at any given clock time, participants had been awake for a longer duration. This can, however, not be the only explanation for the time course of the effects of sleep history because even we compared a Sleep Restriction observation at for example 12∶00 to a Control observation at 16∶00, Alertness in the SR condition was still poorer (see Figure 5A). The time course of the effect of sleep history on performance indicates that a circadian signal can modulate the effects of sleep history on Subjective Alertness, Sustained Attention, and Executive Functions, in accordance with similar observations for Sustained Attention [12]. During the wake maintenance zone when the circadian wake promoting signal is at its crest [48], the brain is relatively protected from the negative effects of sleep deprivation on waking performance. By contrast, in the morning hours, the brain is very susceptible, and all cognitive domains are affected. This circadian modulation of sleep history effects appears strongest for Sustained Attention measures, as reflected in a significant interaction between Condition and Circadian Phase for these measures and in accordance with a previous report [12] in which only Sustained Attention was assessed. The neurochemical basis of this circadian protection of cognition remains to be elucidated, although some suggestions have been made [49].

### Genotype Effects

At baseline, no differences in performance between the *PER3* genotypes were observed. Performance deteriorated following manipulation of sleep homeostasis by both repeated PSD and acute TSD in all three genotypes, but more so in the *PER3^5/5^* homozygotes and in a cognitive-domain-dependent manner [29]. Please note that these differences between the genotypes cannot be explained by differences in sleep timing between the genotypes (see Table S1) because all the events in the laboratory were scheduled relative to habitual sleep times. The more rapid reduction in daytime Subjective Alertness in response to repeated PSD in *PER3^5/5^* homozygotes is a new observation [50], whereas the absence of a Sleep Loss × Genotype interaction on Sustained Attention after both repeated PSD and acute TSD is consistent with previous studies [29], [50], [51]. The exquisite dependency of the expression of the Genotype effect on sleep pressure and circadian phase is in accordance with our previous behavioural [29] and fMRI studies [30] and demonstrates the importance of considering circadian phase and homeostatic sleep pressure in the study of individual differences [24], [25]. In fact, including all performance measures in the analyses of the interaction between homeostatic sleep pressure, circadian phase, and genotype demonstrated that the greatest divergence between individuals and cognitive domains was observed in the early morning hours after sleep deprivation. Thus, sleep deprivation in combination with circadian phase misalignment is a good paradigm to identify individual differences and task specificity in the susceptibility to performance decline.

### Theoretical and Practical Implications

Impairments in performance induced by sleep deprivation have been interpreted within various frameworks, such as the ‘neuropsychological hypothesis’, the ‘vigilance hypothesis’, and the ‘controlled attention hypothesis’ [20]. According to the ‘neuropsychological hypothesis’, sleep deprivation leads to a temporary ‘functional lesion’, particularly in the frontal and prefrontal areas [18], and behavioural deficits in those cognitive domains that rely on these brain areas, such as Executive Functions. Our data clearly indicate that Executive Functions are not more prone to the negative effects of sleep deprivation than Sustained Attention. Furthermore, performance in the n-back tasks that are more difficult and depend heavily on prefrontal mediation are not more affected than simpler versions [52].

The ‘vigilance hypothesis’ states that Sustained Attention is much affected by sleep deprivation and that Sustained Attention is very important to higher aspects of cognition [20]. Although our data show the sensitivity of Sustained Attention tasks to sleep deprivation, they also demonstrate that at the same time, higher aspects of cognition are not very much affected.

By contrast, our data may seem to favour, to some extent, the ‘controlled attention hypothesis’ [53] in which task characteristics related to how engaging they are determine the extent to which they are impaired by sleep deprivation, with the simpler, more monotonous, and less engaging tasks being more affected because in these tasks, ‘top-down’ control which is required for optimal performance is depleted during sleep deprivation. We do not have an independent measure of task engagement, but would note that the two Sustained Attention tasks used in the present study (PVT and SART) are simpler and more monotonous than those tasks assessing Working Memory and Executive Functions (the n-back tasks). In the PVT, participants are exposed to the same stimulus (onset of a time counter) for 10 minutes and need to make a simple response at random intervals ranging between 2 and 10 seconds. Compared to the PVT, the SART is less monotonous because participants are required to decide whether the stimulus displayed is a target or non-target and either withhold or make a response every 1,150 ms for about two minutes. Even more complex and challenging are the verbal n-back tasks in which every 2,500 ms, participants need to update their memory of 1–3 stimuli, compare the current stimulus with the most remote one in their memory, and decide whether it is a match or not, while switching between all of these task components. Due to the differences in the characteristics of these tasks, we postulate that the Working Memory/Executive Function tasks are the most complex, challenging, and engaging tasks, and hence, less affected by sleep deprivation. In fact, some studies have shown that adding an additional item manipulation requirement to a Working Memory task, i.e. increasing the complexity and possibly the degree of participants’ engagement to the task, renders the task less susceptible to the negative effects of sleep deprivation [54].

The observed small deficits after sleep deprivation in more demanding or engaging tasks, the self-reported effort to perform these tasks, and fMRI data [52] all indicate, however, that the top-down executive control of attention required to engage with and perform these task is ultimately affected by sleep deprivation and, in part, forms the basis of inter-individual differences. This theoretical account and our data have a number of practical implications. Most obviously, for young adults, a 6-h sleep opportunity is not sufficient to maintain brain functions. Furthermore, simple measures of Sustained Attention, although sensitive to sleep deprivation, may not predict how well individuals perform on other tasks [55]. Finally, circadian phase is a powerful determinant of the expression of differences in performance between individuals and tasks and identifying the mechanisms by which circadian rhythmicity accomplishes protection against the detrimental effects of sleep loss may provide new tools to improve performance or prevent the deterioration thereof.

Our data show that the extent to which acute TSD leads to deterioration in performance depends on the task domain, prior sleep debt, circadian phase at which performance is assessed, and genetically-determined subject characteristics. Careful consideration of the task characteristics in real life working conditions during night shifts and sustained operations may reduce the risks of performance failures.

### Limitations and Further Research

Our paradigm clearly demonstrates an interaction between the circadian and the homeostatic processes in regulating cognitive performance and implies that Sustained Attention and subjective measures of Alertness and Workload are most affected by both chronic sleep restriction and acute total sleep loss. The contribution of the circadian process was assessed during the acute TSD period. The time course of performance, with a minimum early in the morning and some indications of recovery during the next day clearly indicates the contribution of circadian processes. However, during TSD, both time awake and circadian phase change simultaneously and this paradigm does not allow the examination of the individual contribution of circadian rhythmicity and the sleep homeostat to cognitive performance. The separate contribution of circadian rhythmicity and sleep homeostasis can be assessed in forced desynchrony protocols [10], in which sleep and wake episodes are systematically shifted across the circadian cycle. In future research, multiple cognitive domains should be examined in a forced desynchrony protocol in order to further establish whether the homeostatic and the circadian influence on performance varies across cognitive domains.

In addition, the differences in effect sizes between tasks may be related to the cognitive domain targeted by a specific task, but may, of course, also be related to other aspects of the task such as its duration. Future studies in which time on task effects are analysed across cognitive domains may be of interest.

## Materials and Methods

### Participants

The present study consisted of three phases: (1) telephone screening and screening visit, (2) pre-laboratory field study, and (3) laboratory study. Participants were recruited through flyers, emails, and newspaper and radio advertisements. Out of the 358 individuals who were successfully genotyped after the screening visit, 165 (46.1%) were *PER3^4/4^* homozygotes, 159 (44.4%) were *PER3^4/5^* heterozygotes, and 34 (9.5%) were *PER3^5/5^* homozygotes. The relative prevalence of the three genotypes in this sample is in accordance with previous reports [28], [56], [57]. Thirty-six healthy volunteers were selected and participated in the laboratory study (18 males; mean ± SD of age = 27.6±4.0 years). They were in general good health by medical history, physical examination, and standard biochemistry and haematology tests. They did not suffer from sleep disorders based on self-report questionnaires (Pittsburgh Sleep Quality Index≤5) and a clinical polysomnographical recording conducted during the first night in the laboratory. They reported consuming≤300 mg of caffeine per day and ≤14 units of alcohol per week, and were not smokers or shift workers. They had not travelled across more than one time zone two months before the laboratory phase and had not donated blood six months before. Female volunteers were not pregnant.

This sample consisted of 12 *PER3^4/4^*, 10 *PER3^4/5^*, and 14 *PER3^5/5^* who were matched for age, gender, body mass index (BMI), and ethnicity at the group level (Table S1). The three genotypes also did not differ in their morningness-eveningness preference [58], self-reported sleep quality [59], level of subjective sleepiness [31], [60], personality [61], [62], mental and physical health [63], [64], eating behaviour [65], intelligence [66], [67], and mood state [68] (Table S1). However, genotype-dependent differences were found in actigraphically assessed sleep-wake timing with *PER3^5/5^* individuals going to bed and waking up earlier than *PER3^4/4^*s (Table S1), which is in accordance with a recent large-scale epidemiological study [57]. Three participants (one *PER3^4/5^* and two *PER3^5/5^* individuals) withdrew from the study for personal reasons at various points during the laboratory phase.

Note that this sample was independent of the sample used in our previous behaviour study [29], [51] and also independent of the sample used in our previous fMRI study [30].

### Procedures

#### Genotyping

Genotyping was conducted as described previously [57]. Briefly, a buccal swab was obtained from each participant during the screening visit. Genomic DNA was extracted from the swab (QuickExtract, Epicentre Biotechnologies, Madison, WI) and DNA fragments containing the VNTR polymorphic region were amplified by PCR. PCR fragment length was determined by gel electrophoresis. Genotyping errors were controlled for by inclusion of negative and positive control samples, by repetition of all failed samples, and by repeating a proportion (∼20%) of positive samples. The participants were not informed about their genotype.

#### Assessment of habitual sleep-wake timing

In the two-week period prior to the first laboratory session, participants completed a Karolinska Sleep Diary [69] daily and wore an Actiwatch (wrist-worn Actiwatch L or Actiwatch 4, Cambridge Neurotechnology, Cambridge, UK) which provided continuous assessment of wrist activity levels. Data from the first week, when subjects followed their normal routines, were analysed to determine the habitual sleep-wake timing of each participant. The mid-point of the habitual sleep-wake cycle was calculated and four hours were added to each side to derive the bedtime and wake time of the participant in the following week, as well as during the habituation and baseline nights in the laboratory sessions.

#### Laboratory Study – Design and Rationale

This study was conducted at the Surrey Clinical Research Centre. This study adopted a balanced, cross-over design and consisted of two 12-day, i.e. 11-night, laboratory sessions in which the duration of sleep opportunity was manipulated. The order of experimental conditions was counter-balanced across participants, and the two laboratory sessions were at least 10 days apart. Both laboratory sessions started with a habituation night and a baseline night with 8-h time in bed (TIB; Figure 1A). In the following seven 24-h cycles in the Control condition, TIB was increased by 2 h to 10 h. In young, healthy adults, the asymptotic value of TST is 8.9 h [35], and 8.0–8.7 h is required to maintain performance [70]. With a predicted sleep efficiency of approximately 90%, a 10-h sleep opportunity should be sufficient to maintain performance. In the Sleep Restriction (SR) condition, TIB was reduced by 2 h to 6 h, which is a sleep duration reported by a large segment of the working population [71], [72]. The mid-point of all the sleep episodes, except the recovery sleep episodes, in the laboratory coincided with the mid-point of the participant’s habitual sleep-wake schedule as assessed in the field study. The timing of cognitive performance test batteries and meals during the protocol was adjusted according to the participant’s habitual sleep-wake schedule. The test batteries were administered in each participant’s sound-proof and temperature-regulated room.

During the adaptation, baseline, Control, and SR days, participants stayed indoors and no visitors were allowed. Apart from the time when cognitive performance was assessed, participants spent the majority of their waking hours in the volunteer lounge where they could interact with the staff and other participants, watch TV, listen to music, read, and play board games. In addition to normal indoor lightings, participants were also exposed to indirect natural sunlight through the windows of the volunteer lounge. Three main meals and an evening snack were served each day, and participants had free access to water and fruits (apples and pears). Napping and strenuous physical exercise were not allowed.

Total Sleep Deprivation (TSD): Upon awakening from the last 10-h or 6-h sleep episode, participants stayed awake for 39 h in the Control condition and 41 h in the SR condition under constant routine (CR) conditions modified from [73]. During CR, participants stayed in their bed in their individual room in a semi-recumbent position with light intensity <10 lux at eye level and temperature ranging between 18°C and 22°C. No information related to clock time was provided. Wakefulness was monitored with a video camera as well as continuous electroencephalogram (EEG) and electro-oculogram (EOG). Movement was limited and participants were not allowed to leave their bed. Participants received hourly nutritional drinks (Fortisip: Nutricia, UK) instead of main meals. To meet the daily calorie requirement, the volume for each participant was calculated based on an activity factor of 1.3 and the basal metabolic rate derived from the Schofield equation. Cognitive performance was assessed with a 40-minute test battery every 2 h. For circadian phase assessment, hourly blood samples (7 mL) were collected via an indwelling cannula in the participant’s forearm over a 30-h period during CR starting from 6 and 7 hours upon awakening in the Control and SR conditions respectively. After CR, participants were given a 12-h recovery sleep opportunity. During the entire laboratory sessions, the activities of the participants were closely monitored by the staff.

#### Assessment of cognitive performance

A cognitive performance test battery was administered 7–8 times in total on the first two days of each laboratory session to familiarize participants with the cognitive tasks and minimize any effect of practice and learning on performance on subsequent days. On the baseline, and each Control and SR day, the test battery was administered five times which were evenly distributed across the waking episodes. During the TSD period, the test battery was presented every 2 h starting from two hours after scheduled wake time. The test battery were administered on identical computers with screen refresh rates of 60 Hz and running Active X, C#, and Exactics code to control stimulus presentation and its timing, and response detection and timing. Each test battery lasted for approximately 40 minutes. In the main text of this paper, we report the data from the Karolinska Sleepiness Scale (KSS) [31], the Psychomotor Vigilance Task (PVT) [32], Sustained Attention Response Task (SART) [33] , and verbal 1-, 2-, and 3-back tasks [29], [74]. The tasks were presented in one of the three orders fixed for each participant (Table S7). In the supplemental figures, we also report data in relation to Temporal and Motor Control, Subjective Workload measures, as well as Affect.

The KSS assessed level of subjective sleepiness. Participants were required to rate how sleepy they were at the beginning and the end of the test battery on a 9-point Likert scale (1: very alert; 9: very sleepy, great effort to keep awake). In the main text, we focused on the first KSS score collected in the test battery.

The PVT assessed level of Sustained Attention. A counter in the middle of the computer screen started counting at random intervals which varied from 2,000 ms to 10,000 ms, and participants were required to respond with a mouse click as quickly as possible. In order to minimize the number of microsleep during this task in the TSD period, a beep was presented to alert the participants if no response was detected 6,000 ms after stimulus presentation. This task lasted for 10 minutes. The inverse of the reaction time of the 10% slowest responses and the number of lapses, i.e. responses with reaction time >500 ms, were used to indicate level of Sustained Attention since these measures are considered to be sensitive to both chronic and acute sleep deprivation [36]. In accordance with recommendations from [75], the number of lapses was first transformed (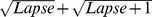) in all the analyses.

Sustained Attention was also assessed with the SART. A series of numbers from 0 to 9 was presented to the participants who were required to make a mouse click in every trial except when the target number, i.e. 8, was presented when they needed to withhold their response. The target:distractor ratio was 15∶85, and the inter-stimulus interval was 900 ms. We first derived the hit rate (the number of non-target trials the participants made a response × 100/85) and the false alarm rate (the number of target trials the participants responded to × 100/15). We then computed A’ to indicate the participant’s ability to discriminate between target and distractor trials by using the formula provided in the next section.

The verbal n-back tasks assessed participants’ Working Memory/Executive Functions. Verbal 1-back was always presented to the participants first, followed by 2- and 3-back. Participants were shown one of the letters (B, C, D, F, G, H, J, K, and M) for 500 ms and required to compare it with the letter presented n trials before. The inter-stimulus interval was 2,000 ms. The match:mismatch trial ratio was 8∶24. We first computed the hit rate (hit = number of correct match trials × 100/number of match trials) and the false alarm rate (fa = number of incorrect mismatch trials × 100/number of mismatch trials). We then computed non-parametric measures of sensitivity (A′) and response bias (B″_D_) which were introduced by [76] and popularised by [77], [78].
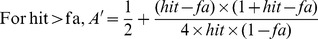


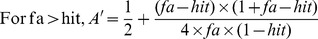


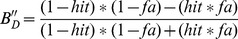



A′ is one of the discriminability measures in signal detection theory. It is a non-parametric analogue of the more widely used d’ and can still be derived when the hit or false alarm rate is 0 or 1. A′ ranges from 0 to 1, with 0.5 suggesting chance performance. Its corresponding bias measure is B″_D_ which indicates whether participants (a) tended to provide a ‘Yes’ response and indicate the stimuli matched, i.e. they were liberal and more likely to detect matches when they were actually present (B″_D_ <0), (b) tended to provide a ‘No’ response and indicate the stimuli did not match, i.e. they were conservative and less likely to detect matches when they were actually present (B″_D_ >0), or (c) were neutral in their tendency to provide ‘Yes’ and ‘No’ responses (B″_D = _0).

After each verbal n-back task, the participants were asked to report their subjective ratings of the energy, cognitive demand, mental effort, and physical effort required to perform the task on a Visual Analogue Scale. We also measured the participants’ mood with the Positive and Negative Affect Scale [68]. Motor Control was assessed with the Pursuit Tracking Task [79], [80], and Temporal Control was indicated by their performance in the Fixed/Random Interval Repetition Tasks [81].

#### Melatonin assays and assessment of circadian phase

The hourly blood samples collected during CR were centrifuged (15 min, 1620 × *g*, and 4°C) within 20 minutes upon collection to separate the plasma which was then stored at −20°C until assay. Plasma melatonin concentration was assessed with radioimmunoassay (Stockgrand, Guildford, Surrey, United Kingdom). The limit of detection was 3.4 pg/mL. The interassay coefficients of variation were 21.9% at 8.5±1.9 pg/mL, 13.4% at 36.6±4.9 pg/mL, 13.5% at 81.0±10.9 pg/mL, and 11.7% at 123.5±14.0 pg/mL. Melatonin is considered a reliable marker of circadian phase and extensive comparisons of the robustness and sensitivity of various melatonin phase markers are available [82]. To determine the dim light melatonin onset (DLMO), for each participant in each condition, we first established the baseline melatonin level using the median melatonin concentration of the first five samples collected as well as the maximum level using the median of the highest three concentrations so as to avoid local maxima or minima. The time of the DLMO was derived with linear interpolation between the melatonin sample just below and the one just above 25% of the difference between the baseline and the maximum at the rising limb of the melatonin profile. Similarly, the dim light melatonin offset (DLMOff) was determined at the declining limb of the profile. The dim light melatonin mid-point was the average of the DLMO and the DLMOff. The onset, offset, and mid-point of dim light melatonin were also assessed at a 50% level. Furthermore, in order to derive the amplitude of the melatonin profile and the time of the melatonin peak, for each participant in each condition, we fitted the melatonin data with a sinusoidal function:
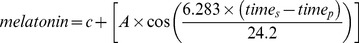
where c = constant, A = fitted amplitude, time_s_ = sampling time, and time_p_ = time of fitted melatonin peak. 24.2 is the average period of the human circadian pacemaker when assessed under conditions in which the effects of the light-dark cycle and feedback from the sleep-wake cycle are minimized [83]. We used this period because we assumed that under the CR condition, the circadian pacemaker oscillates at its intrinsic period [84].

#### Polysomnography

EEG signals in all sleep episodes were recorded using a 10-channel EEG montage (Fz-A2, Cz-A1, F3-A2, F4-A1, C3-A2, C4-A1, P3-A2, P4-A1, O1-A2, and O2-A1) according to the 10-20 system. T3-A2 and T4-A1 were added to the montage during those nights after participants had performed a declarative memory task, which will be reported elsewhere. Eye movement, muscle tone, and heart rate were recorded through left and right EOG, submental EMG, and ECG electrodes, which were respectively referenced to A2 and A1. The ground and common reference electrodes were placed at FPz and Pz, respectively. Participants also wore a thoracic band, a nasal airflow sensor, a microphone, and leg electrodes during the habituation night in the first laboratory session to monitor any sign of sleep-related breathing problems and periodic leg movements.

The EEG, EOG, and EMG signals were recorded on Siesta 802 devices (Compumedics, Abbotsford, Victoria, Australia). The sampling rate and the storage was 256 Hz. The low-pass filter was set at 70 Hz and the high-pass filter was set at 0.3 Hz. Electrode impedance was kept below 5 kΩ. Sleep staging was performed according to the Rechtschaffen and Kales criteria [85] and the scorer was blind to genotype.

### Ethics

This protocol was approved by the Institutional Review Board of the Air Force Research Laboratory and received a favourable opinion from the University of Surrey Ethics Committee. It was conducted in accordance with the principles of the Declaration of Helsinki. All the participants provided written informed consent after receiving a detailed explanation of the aims and procedures of the study.

### Statistical Methods

Statistical analyses were performed with SAS 9.1 (SAS Institute, Cary, NC). We used a general linear mixed model with PROC MIXED to determine the effects of Genotype (*PER3^4/4^*/*PER3^4/5^*/*PER3^5/5^*), Condition (Control/SR), Session (first/second laboratory session), and the Genotype × Condition interaction, as fixed model effects, on DLMO and other circadian phase markers, with the Subject effect as a random factor. For the performance data, we also examined the effects of Day (from baseline to the second day of TSD) as a repeated effect with a spatial power variance-covariance matrix being specified. For the performance data during the TSD period, we first aligned the data to the melatonin rhythm, and instead of the Day effect, we examined the Circadian effect (from DLMO −12 h to DLMO +20 h; 4-h bins) as a repeated effect with a first-order autoregressive variance-covariance matrix being specified. Differences of least square means were used to determine significant differences among the *PER3* genotypes and between the two conditions at *P*<0.05.

Sleep data during the habituation night were not included in the analyses. Performance data collected on the arrival and habituation days were also not included in the analyses to minimize the effect of learning and practice on the results. Due to technical problems and drop-outs, 2.8% of the performance data, 8.6% of the PSG records, and 6.4% of the blood samples could not be included in the analyses.

Effect sizes were indicated by Cohen’s *f^2^* [34], [41]:
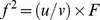
where *u* and *v* are respectively the numerator and denominator degrees of freedom of the *F* statistic used to determine the corresponding main or interaction effect in the general linear mixed model analysis.

In addition to using effect sizes to quantify the effects of repeated PSD on Sustained Attention and Executive Functions, we also compared the magnitude of change in performance in the PVT and the verbal 3-back task from the baseline day to the end of the partial sleep deprivation period (expressed as the performance on D6 in the SR condition divided by the performance on the baseline day) with the change in performance across the same days in the Control condition. For the effect of acute TSD, we did a similar comparison between the change in performance from the first to the second day of the TSD period between the two conditions (expressed as performance on TD2/performance on TD1).

## Supporting Information

Figure S1
**Effect of sleep history on Sustained Attention and Working Memory.** ***(A)*** Sustained Attention indicated by the number of lapses (reaction time >500 ms) in the Psychomotor Vigilance Task. Analysis was performed after transformation (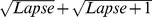). ***(B)*** Bias (B”_D_) of Working Memory tasks with increasing executive load (verbal 1- to 2- to 3-back). B = Baseline, D1–D6 = the days during the Sleep Restriction/Control condition. TSD = Total Sleep Deprivation. TD1 = first day of total sleep deprivation. TN1 = night of total sleep deprivation, TD2 = second day of total sleep deprivation. In all panels, the least square means and standard errors estimated with PROC MIXED in SAS are plotted. Asterisks indicate the significance of the contrast between conditions (***P<0.001, **P<0.01, and *P<0.05). Open circles = Control condition; filled circles = Sleep Restriction condition.(TIF)

Figure S2 **Comparison of effect sizes of sleep restriction for Subjective Alertness, Sustained Attention, and Working Memory throughout the protocol (B - TD2).**
(TIF)

Figure S3 **Effect size of repeated partial sleep deprivation on all performance measures from the first to the sixth day of sleep loss.** PANAS = Positive and Negative Affect Scale; PTT = Pursuit Tracking Task; SD = standard deviation; ED = Euclidean distance; RIR = Random Interval Repetition task; FIR = Fixed Interval Repetition task; RT = reaction time; B”_D_ = bias; SART: Sustained Attention to Response Task; PVT = Psychomotor Vigilance Task; KSS = Karolinska Sleepiness Scale (KSS1 and KSS2 were respectively administered at the beginning and the end of the test battery); *f^2^* = implied effect size.
(TIF)

Figure S4 **Comparison of effect sizes for Subjective Alertness, Sustained Attention, Working Memory and the corresponding subjective ratings, Temporal and Motor Control, and Affect.** **(** ***A*** **)** Effect size of repeated partial sleep deprivation. It was assessed by comparing performance during D5 and D6 between conditions. Subjective Alertness, Sustained Attention, and the Subjective Workload of the Working Memory tasks were the most affected by repeated partial sleep deprivation. **(** ***B*** **)** Effect size of acute total sleep deprivation. It was assessed by comparing performance on TD1 to performance on TD2 across conditions. Subjective Alertness and Sustained Attention were the most vulnerable to the impairing effects of acute total sleep deprivation. Horizontal lines indicate cut-offs for small, medium, and large effect sizes. Refer to Figure S3 for the explanations of the task and variable abbreviations.
(TIF)

Figure S5
**Effect of** ***PER3*** **genotype on performance during partial sleep deprivation and subsequent total sleep deprivation.** Time course of ***(A)*** Sustained Attention as indicated by the speed of the 10% slowest responses and the number of lapses in the Psychomotor Vigilance Task (PVT) and A’ in the Sustained Attention to Response Task (SART), and ***(B)*** Working Memory as indicated by B”_D_ in the verbal 3-back task in *PER3^4/4^*, *PER3^4/5^*, and *PER3^5/5^* individuals. Analysis on PVT lapses was performed after transformation (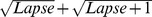). The least square means and standard errors estimated with PROC MIXED in SAS are plotted. Asterisks indicate the significance of the contrast between conditions (***P<0.001, **P<0.01, and *P<0.05). Open circles = Control condition; filled circles = Sleep Restriction condition.(TIF)

Figure S6 **Comparison of the effect sizes for the Genotype × Condition interaction for Subjective Alertness, Sustained Attention, and Working Memory throughout the protocol (B-TD2).**
(TIF)

Figure S7 **Comparison of effect sizes of acute total sleep deprivation for Subjective Alertness, Sustained Attention, and Working Memory in the** ***PER3*** **genotypes.** In all the *PER3* genotypes, acute total sleep deprivation (assessed by comparing performance on TD1 to performance on TD2 across conditions) had greater impairing effects on Subjective Alertness and Sustained Attention than on Working Memory/Executive Functions.
(TIF)

Figure S8 **Effect of sleep history on the circadian rhythm of plasma melatonin.** Repeated partial sleep restriction led to a significant delay in the melatonin rhythm as assessed by the dim light melatonin onset (DLMO; 25%). The sleep period in the Sleep Restriction (SR) and the Control conditions is respectively indicated by the dark and the light gray areas. The dash and the solid vertical lines respectively indicate the DLMO in the SR and the Control conditions. Phase angle refers to the difference between DLMO and the midpoint of the scheduled wake episode before the total sleep deprivation period.
(TIF)

Figure S9 **Effects size of prior partial sleep deprivation on performance during total sleep deprivation calculated separately per 4-h circadian melatonin bins for Subjective Alertness, Sustained Attention, and Working Memory.**
(TIF)

Figure S10 **Circadian modulation of effect size on each of the 52 performance measures during total sleep deprivation calculated separately per 4-h circadian melatonin bins.** ***(A)*** Effects size of prior partial sleep deprivation on performance. ***(B)*** Effect size of the interaction of genotype and prior partial sleep deprivation on performance. Refer to Figure S3 for the explanations of the task and variable abbreviations.
(TIF)

Table S1 **Characteristics of** ***PER3^4/4^*** **,** ***PER3^4/5^*** **, and** ***PER3^5/5^*** **participants (mean ± standard deviation).**
(DOC)

Table S2 **Results of a general linear mixed model examining the effects of Condition (Sleep Restriction vs. Control) and Day (from baseline to the second day of total sleep deprivation) on performance.**
(DOC)

Table S3 **Effects of repeated partial and acute total sleep deprivation on performance.**
(DOC)

Table S4 **Results of a general linear mixed model examining the effects of Genotype, Condition (Sleep Restriction vs. Control), and Day (from baseline to the second day of total sleep deprivation) on performance.**
(DOC)

Table S5 **Circadian phase markers of the** ***PER3^4/4^*** **,** ***PER3^4/5^*** **, and** ***PER3^5/5^*** **participants.**
(DOC)

Table S6 **Results of a general linear mixed model examining the effects of Condition (Sleep Restriction vs. Control) and Circadian Phase (between DLMO 12 h to DLMO+20 h) on performance.**
(DOC)

Table S7 **The three orders of the cognitive tasks included in the test battery.**
(DOC)
