# Supplementary figures and images for: Effects of Partial and Acute Total Sleep Deprivation on Performance across Cognitive Domains, Individuals and Circadian Phase
Source: PLoS One. 2012 Sep 24;7(9):e45987. doi: 10.1371/journal.pone.0045987 (PMC3454374; doi:10.1371/journal.pone.0045987)

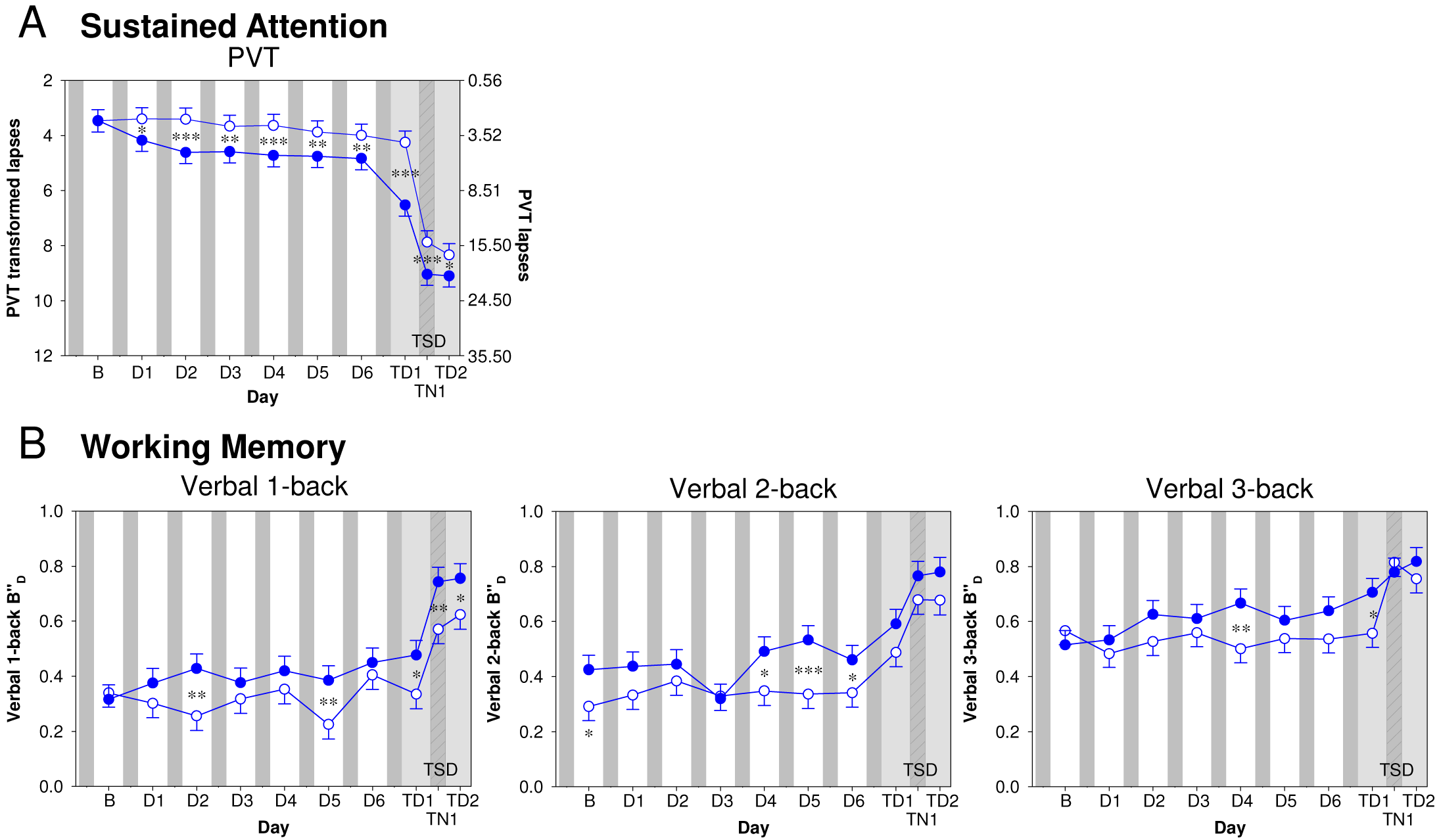

Supplement: Figure S1 — Effect of sleep history on Sustained Attention and Working Memory. (A) Sustained Attention indicated by the number of lapses (reaction time >500 ms) in the Psychomotor Vigilance Task. Analysis was performed after transformation (). (B) Bias (B”D) of Working Memory tasks with increasing executive load (verbal 1- to 2- to 3-back). B = Baseline, D1–D6 = the days during the Sleep Restriction/Control condition. TSD = Total Sleep Deprivation. TD1 = first day of total sleep deprivation. TN1 = night of total sleep deprivation, TD2 = second day of total sleep deprivation. In all panels, the least square means and standard errors estimated with PROC MIXED in SAS are plotted. Asterisks indicate the significance of the contrast between conditions (***P<0.001, **P<0.01, and *P<0.05). Open circles = Control condition; filled circles = Sleep Restriction condition. (TIF) [file pone.0045987.s001.tif]

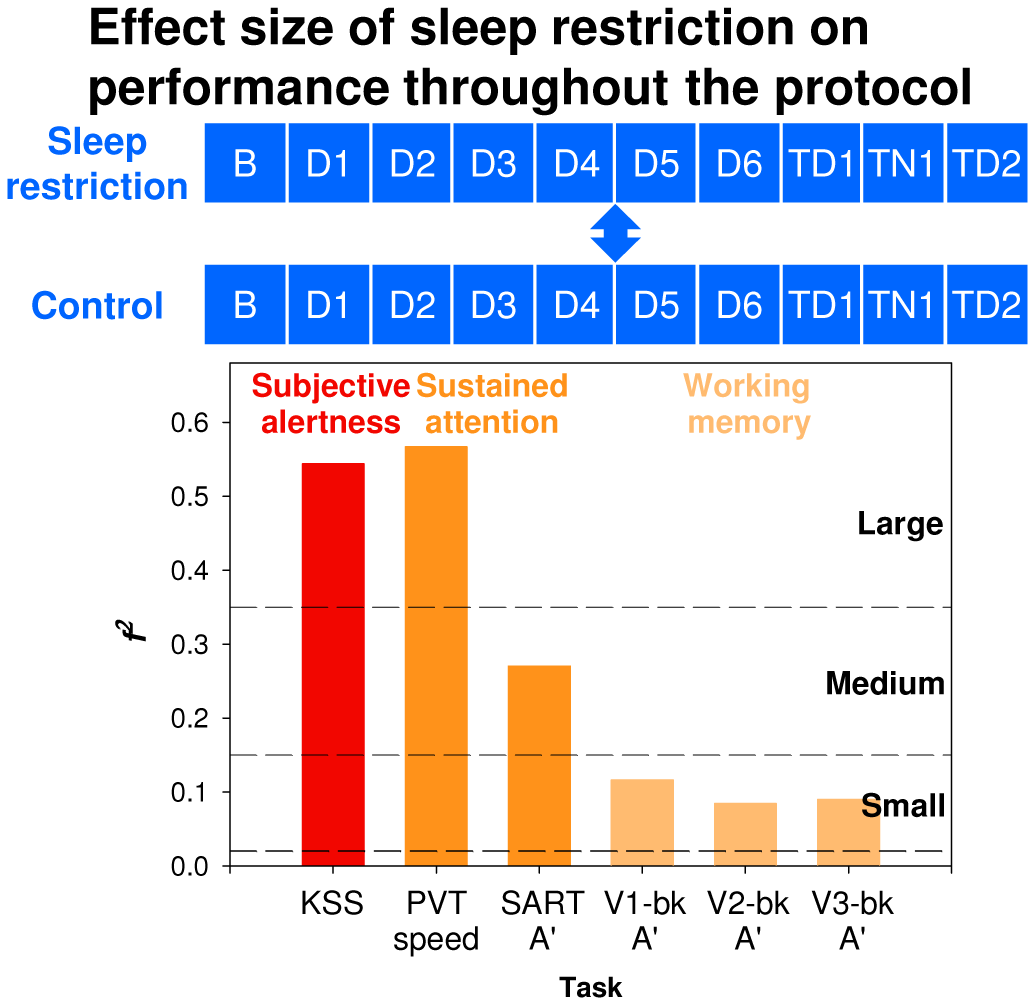

Supplement: Figure S2 — Comparison of effect sizes of sleep restriction for Subjective Alertness, Sustained Attention, and Working Memory throughout the protocol (B - TD2). (TIF) [file pone.0045987.s002.tif]

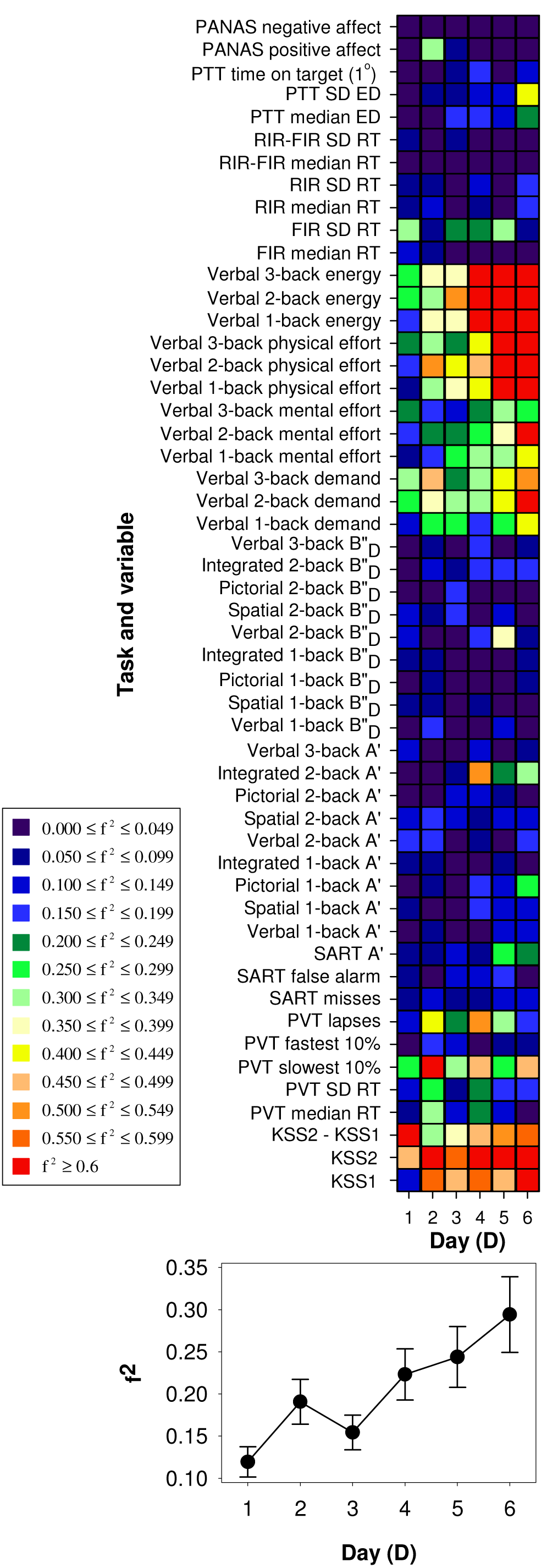

Supplement: Figure S3 — Effect size of repeated partial sleep deprivation on all performance measures from the first to the sixth day of sleep loss. PANAS = Positive and Negative Affect Scale; PTT = Pursuit Tracking Task; SD = standard deviation; ED = Euclidean distance; RIR = Random Interval Repetition task; FIR = Fixed Interval Repetition task; RT = reaction time; B”D = bias; SART: Sustained Attention to Response Task; PVT = Psychomotor Vigilance Task; KSS = Karolinska Sleepiness Scale (KSS1 and KSS2 were respectively administered at the beginning and the end of the test battery); f2 = implied effect size. (TIF) [file pone.0045987.s003.tif]

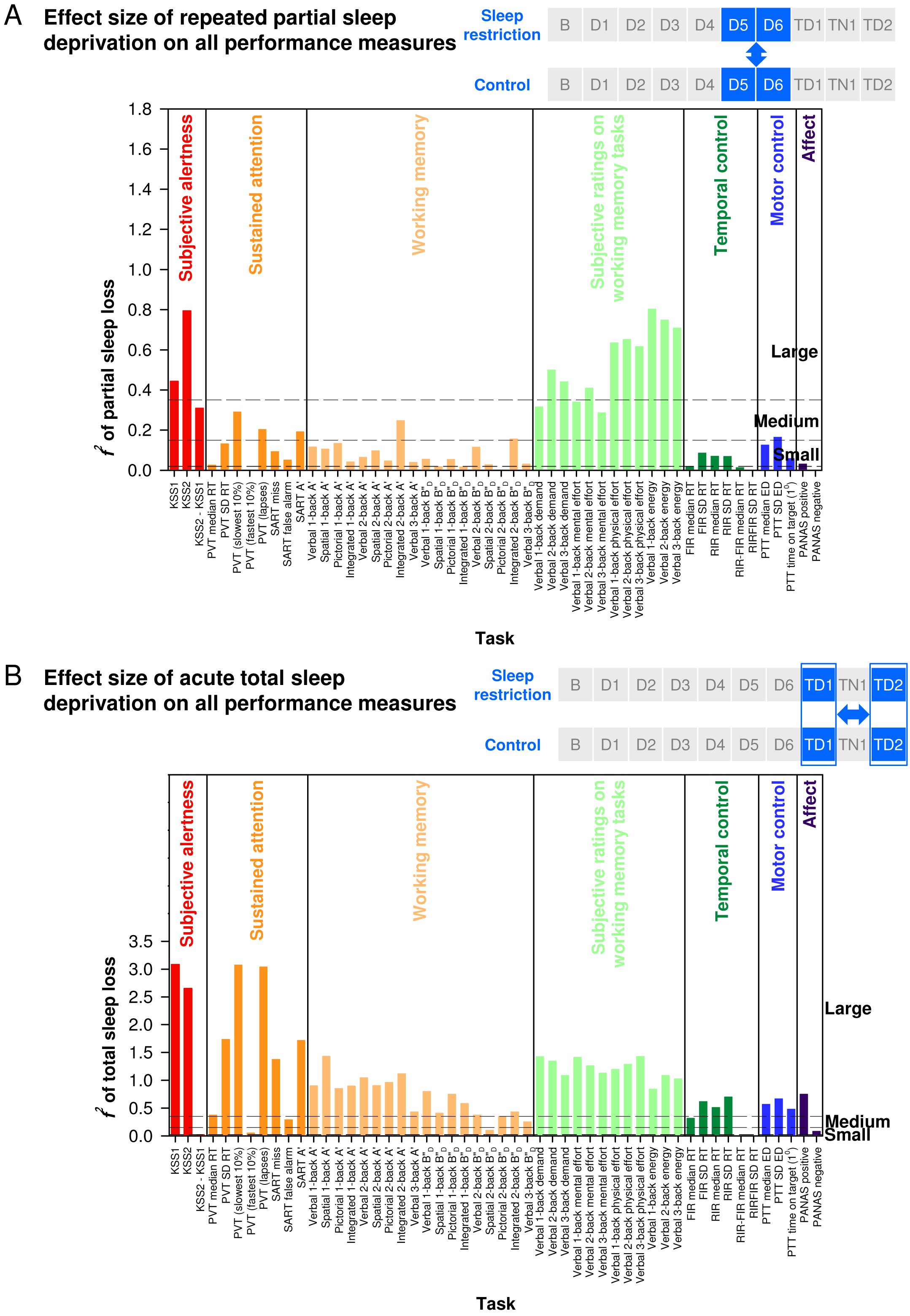

Supplement: Figure S4 — Comparison of effect sizes for Subjective Alertness, Sustained Attention, Working Memory and the corresponding subjective ratings, Temporal and Motor Control, and Affect. ( A ) Effect size of repeated partial sleep deprivation. It was assessed by comparing performance during D5 and D6 between conditions. Subjective Alertness, Sustained Attention, and the Subjective Workload of the Working Memory tasks were the most affected by repeated partial sleep deprivation. ( B ) Effect size of acute total sleep deprivation. It was assessed by comparing performance on TD1 to performance on TD2 across conditions. Subjective Alertness and Sustained Attention were the most vulnerable to the impairing effects of acute total sleep deprivation. Horizontal lines indicate cut-offs for small, medium, and large effect sizes. Refer to Figure S3 for the explanations of the task and variable abbreviations. (TIF) [file pone.0045987.s004.tif]

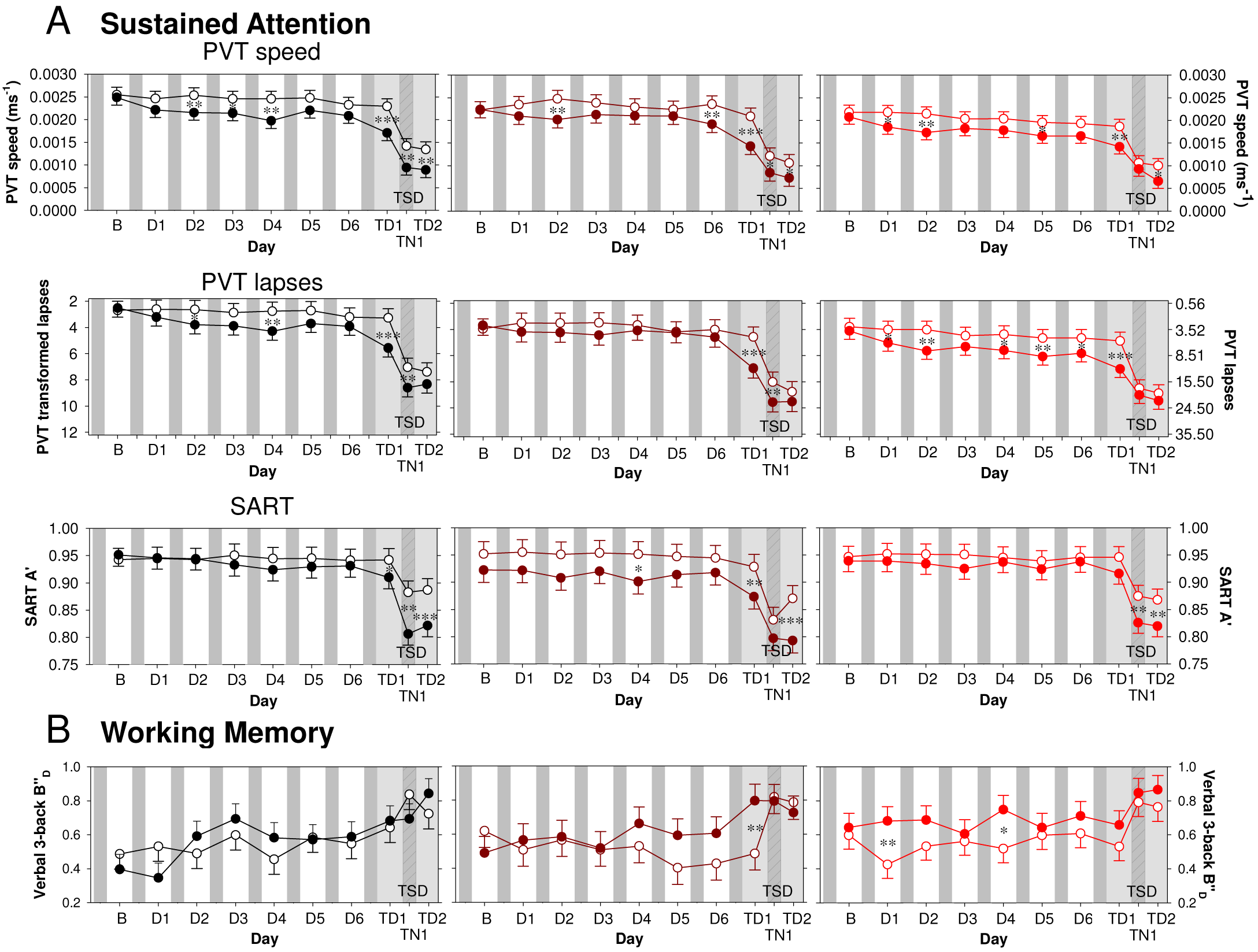

Supplement: Figure S5 — Effect of PER3 genotype on performance during partial sleep deprivation and subsequent total sleep deprivation. Time course of (A) Sustained Attention as indicated by the speed of the 10% slowest responses and the number of lapses in the Psychomotor Vigilance Task (PVT) and A’ in the Sustained Attention to Response Task (SART), and (B) Working Memory as indicated by B”D in the verbal 3-back task in PER34/4, PER34/5, and PER35/5 individuals. Analysis on PVT lapses was performed after transformation (). The least square means and standard errors estimated with PROC MIXED in SAS are plotted. Asterisks indicate the significance of the contrast between conditions (***P<0.001, **P<0.01, and *P<0.05). Open circles = Control condition; filled circles = Sleep Restriction condition. (TIF) [file pone.0045987.s005.tif]

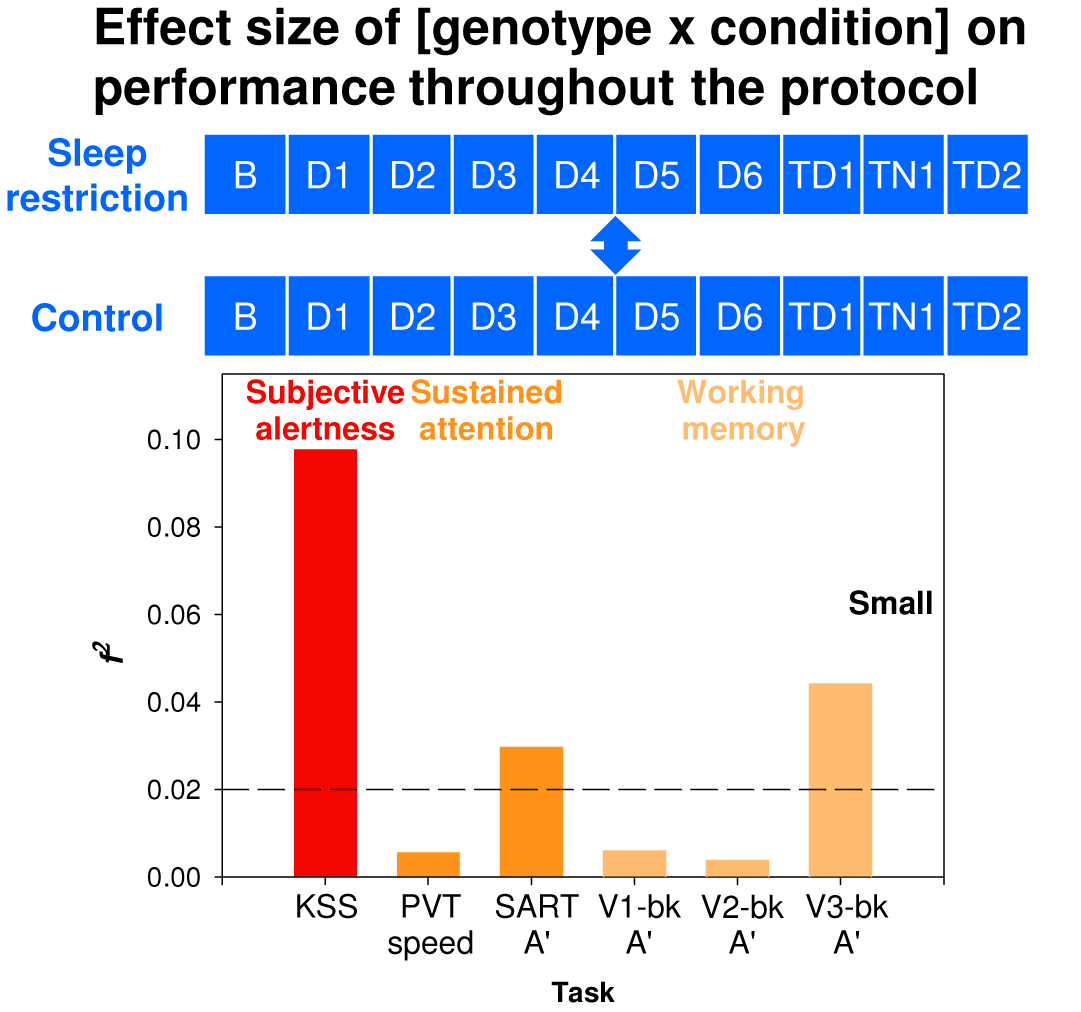

Supplement: Figure S6 — Comparison of the effect sizes for the Genotype × Condition interaction for Subjective Alertness, Sustained Attention, and Working Memory throughout the protocol (B-TD2). (TIF) [file pone.0045987.s006.tif]

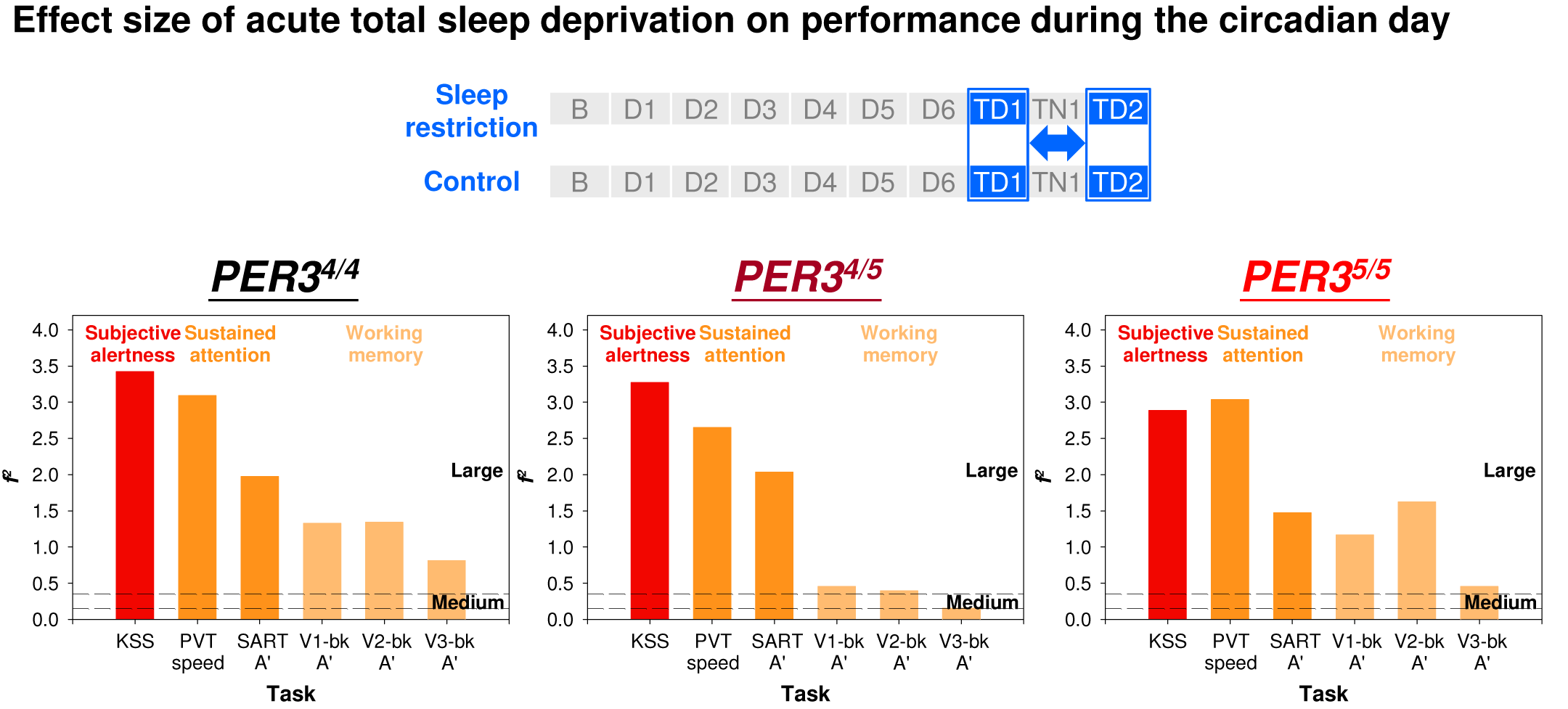

Supplement: Figure S7 — Comparison of effect sizes of acute total sleep deprivation for Subjective Alertness, Sustained Attention, and Working Memory in the PER3 genotypes. In all the PER3 genotypes, acute total sleep deprivation (assessed by comparing performance on TD1 to performance on TD2 across conditions) had greater impairing effects on Subjective Alertness and Sustained Attention than on Working Memory/Executive Functions. (TIF) [file pone.0045987.s007.tif]

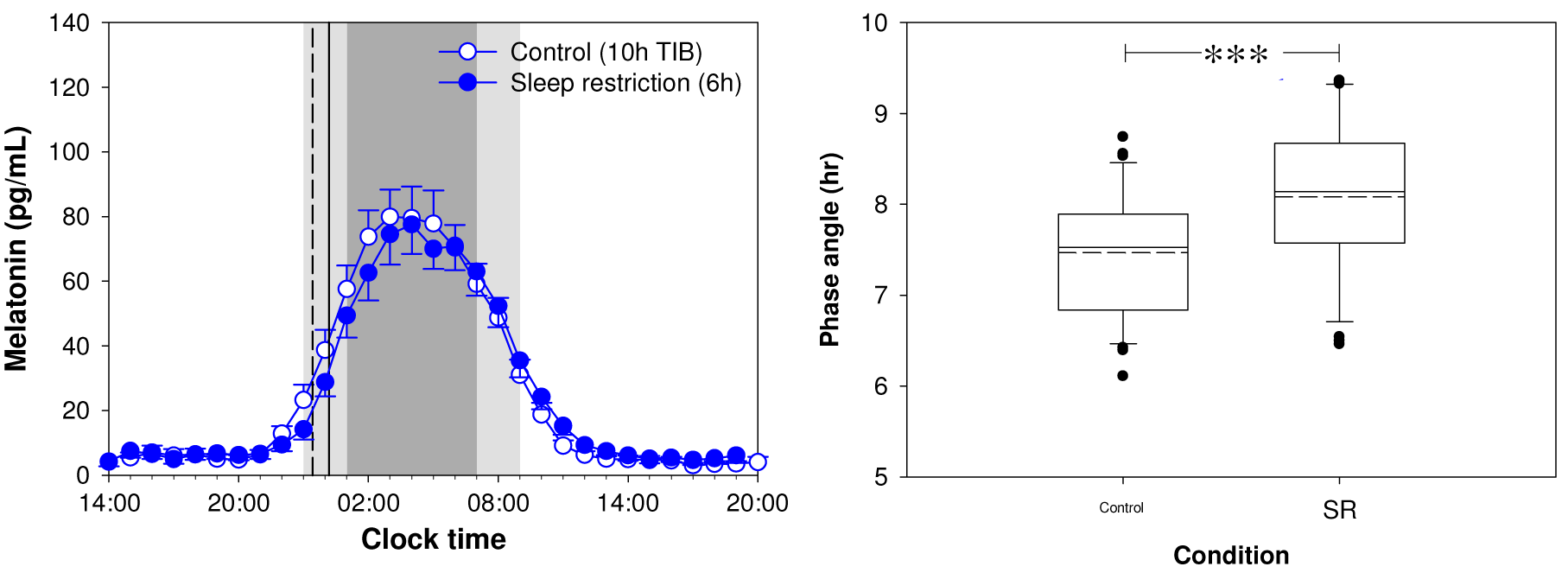

Supplement: Figure S8 — Effect of sleep history on the circadian rhythm of plasma melatonin. Repeated partial sleep restriction led to a significant delay in the melatonin rhythm as assessed by the dim light melatonin onset (DLMO; 25%). The sleep period in the Sleep Restriction (SR) and the Control conditions is respectively indicated by the dark and the light gray areas. The dash and the solid vertical lines respectively indicate the DLMO in the SR and the Control conditions. Phase angle refers to the difference between DLMO and the midpoint of the scheduled wake episode before the total sleep deprivation period. (TIF) [file pone.0045987.s008.tif]

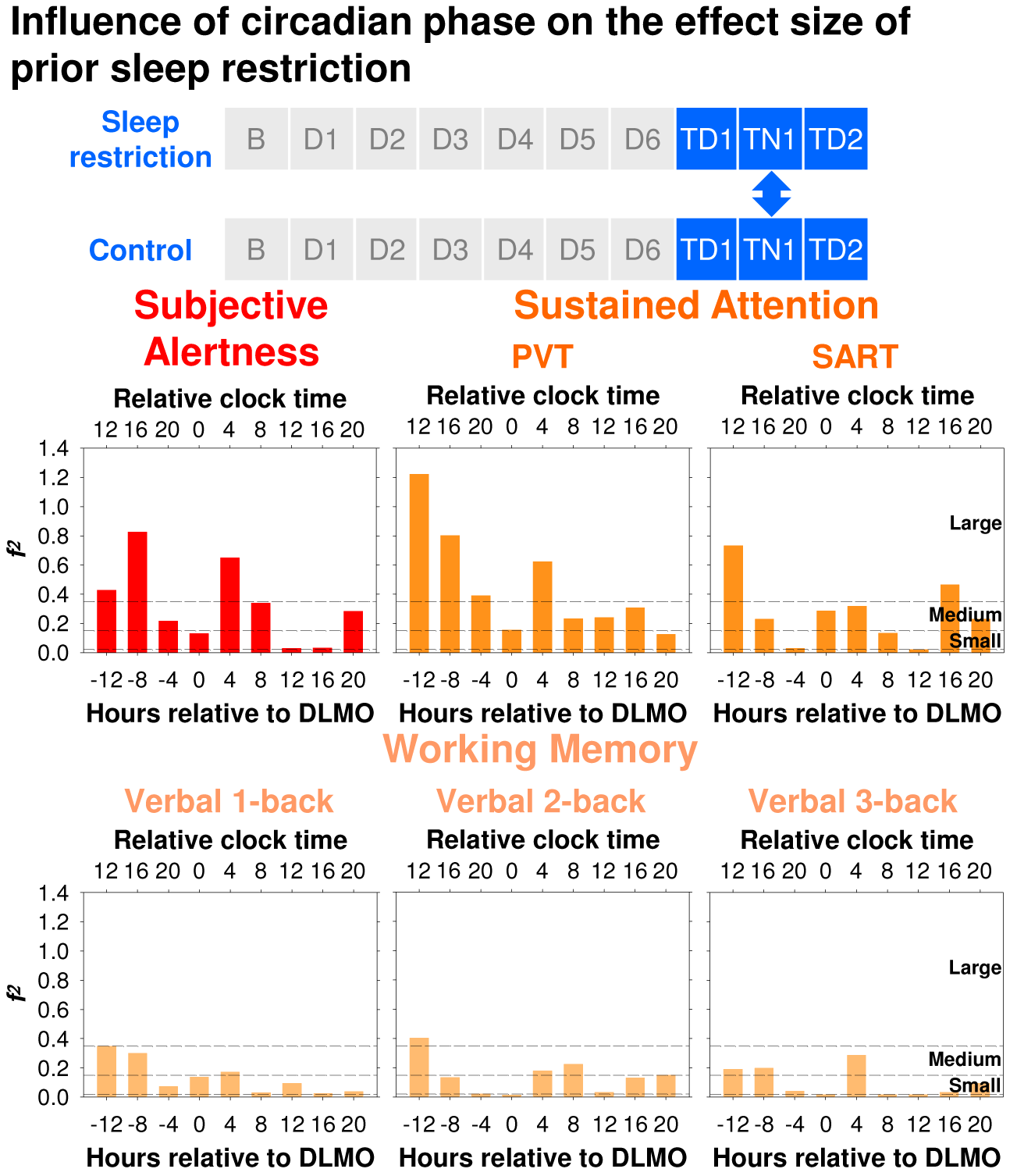

Supplement: Figure S9 — Effects size of prior partial sleep deprivation on performance during total sleep deprivation calculated separately per 4-h circadian melatonin bins for Subjective Alertness, Sustained Attention, and Working Memory. (TIF) [file pone.0045987.s009.tif]

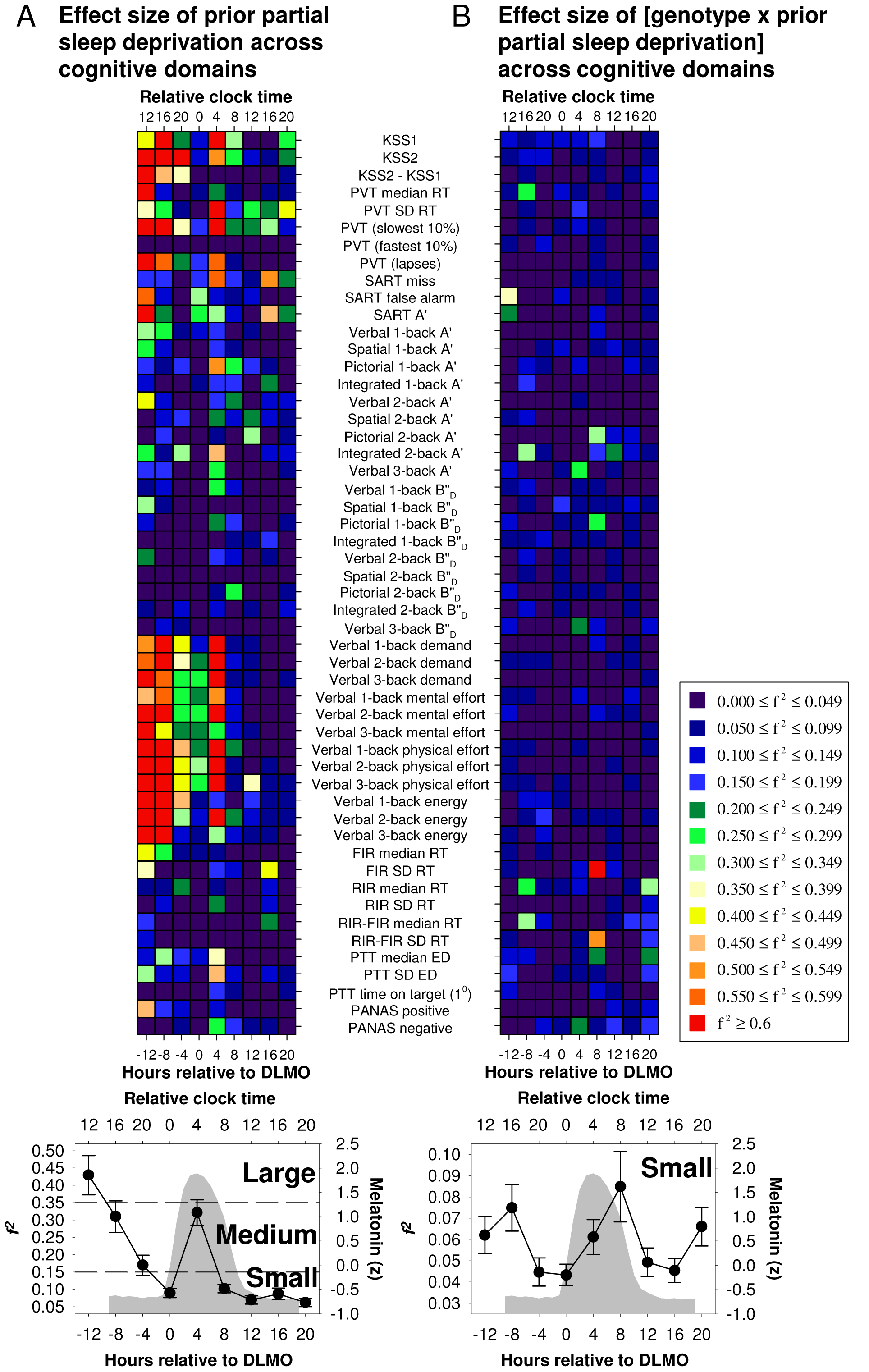

Supplement: Figure S10 — Circadian modulation of effect size on each of the 52 performance measures during total sleep deprivation calculated separately per 4-h circadian melatonin bins. (A) Effects size of prior partial sleep deprivation on performance. (B) Effect size of the interaction of genotype and prior partial sleep deprivation on performance. Refer to Figure S3 for the explanations of the task and variable abbreviations. (TIF) [file pone.0045987.s010.tif]
