# Supplementary material for: Effects of Partial and Acute Total Sleep Deprivation on Performance across Cognitive Domains, Individuals and Circadian Phase
Source: PLoS One. 2012 Sep 24;7(9):e45987. doi: 10.1371/journal.pone.0045987 (PMC3454374; doi:10.1371/journal.pone.0045987)
Supplement: Table S1 — Characteristics of PER34/4 , PER34/5 , and PER35/5 participants (mean ± standard deviation). (DOC) [file pone.0045987.s011.doc]

**Table S1** Characteristics of *PER3^4/4^*, *PER3^4/5^*, and *PER3^5/5^* participants (mean ± standard deviation)

| **Measure** | ***PER3^4/4^*** | ***PER3^4/5^*** | ***PER3^5/5^*** |  |
| --- | --- | --- | --- | --- |
| **n** | 12 | 10 | 14 | - |
| **Demographics** |  |  |  |  |
| Age | 27.08 ± 4.36 | 27.50 ± 3.54 | 28.14 ± 4.22 | *F_2,33_* = 0.22; *P* = 0.80 |
| Gender (male : female) | 6 : 6 | 4 : 6 | 8 : 6 | *X^2^_2_* = 0.69; *P* = 0.71 |
| Body mass index | 22.95 ± 2.68 | 23.84 ± 2.88 | 24.67 ± 2.43 | *F_2,33_* = 1.36; *P* = 0.27 |
| Ethnicity (Caucasian: Others) | 9 : 3 | 9 : 1 | 10 : 4 | *X^2^_2_* = 1.24; *P* = 0.54 |
| **Sleep** |  |  |  |  |
| Horne-Östberg Questionnaire | 46.25 ± 9.49 | 54.20 ± 10.95 | 52.07 ± 6.32 | *F_2,33_* = 2.46; *P* = 0.10 |
| Pittsburgh Sleep Quality Index | 2.50 ± 1.24 | 2.40 ± 1.58 | 2.43 ± 1.28 | *F_2,33_* = 0.02; *P* = 0.98 |
| Epworth Sleepiness Scale | 5.92 ± 4.36 | 5.00 ± 3.56 | 3.57 ± 2.34 | *F_2,33_* = 1.52; *P* = 0.23 |
| Karolinska Sleepiness Scale | 3.58 ± 1.56 | 3.30 ± 1.16 | 2.93 ± 1.33 | *F_2,33_* = 0.75; *P* = 0.48 |
| Insomnia Severity Index | 3.17 ± 3.76 | 2.40 ± 3.89 | 1.64 ± 1.45 | *F_2,33_* = 0.78; *P* = 0.47 |
| Habitual bedtime (actigraphy)* | 00:30 ± 00:17† | 23:54 ± 00:07 | 23:36 ± 00:15† | *F_2,33_* = 3.76; ***P* = 0.03** |
| Habitual wake time (actigraphy)* | 08:52 ± 00:24† | 08:27 ± 00:17 | 07:40 ± 00:17† | *F_2,33_* = 3.56; ***P* = 0.04** |
| Habitual time in bed (actigraphy)* | 08:22 ± 00:12 | 08:34 ± 00:14 | 08:04 ± 00:10 | *F_2,33_* = 1.66; *P* = 0.21 |
| **Personality** |  |  |  |  |
| Openness (BFI) | 39.00 ± 7.57 | 40.30 ± 3.83 | 37.86 ± 6.89 | *F_2,33_* = 0.42; *P* = 0.66 |
| Conscientiousness (BFI) | 34.42 ± 5.71 | 35.80 ± 8.05 | 35.07 ± 3.58 | *F_2,33_* = 0.16; *P* = 0.86 |
| Extraversion (BFI) | 27.25 ± 6.93 | 31.00 ± 6.27 | 28.31 ± 6.36 | *F_2,33_* = 0.94; *P* = 0.40 |
| Agreeableness (BFI) | 35.92 ± 4.64 | 37.80 ± 5.67 | 37.79 ± 4.39 | *F_2,33_* = 0.60; *P* = 0.56 |
| Neuroticism (BFI) | 18.08 ± 4.96 | 15.78 ± 4.44 | 15.29 ± 5.38 | *F_2,33_* = 1.09; *P* = 0.35 |
| Behavioural inhibition (BIS/BAS) | 17.00 ± 4.41 | 19.10 ± 5.70 | 18.25 ± 3.98 | *F_2,33_* = 0.56; *P* = 0.58 |
| Behavioural activation – reward (BIS/BAS) | 6.92 ± 2.57 | 7.10 ± 2.28 | 8.77 ± 2.20 | *F_2,33_* = 2.31; *P* = 0.12 |
| Behavioural activation – drive (BIS/BAS) | 8.67 ± 2.42 | 6.70 ± 2.67 | 8.71 ± 2.16 | *F_2,33_* = 2.50; *P* = 0.10 |
| Behavioural activation – fun seeking (BIS/BAS) | 6.67 ± 1.87 | 6.60 ± 2.27 | 7.50 ± 2.31 | *F_2,33_* = 0.68; *P* = 0.51 |
| **Health** |  |  |  |  |
| General Health Questionnaire | 7.83 ± 1.99 | 6.33 ± 2.69 | 7.50 ± 2.07 | *F_2,33_* = 1.26; *P* = 0.30 |
| Physical function (SF36) | 29.92 ± 0.29 | 29.90 ± 0.32 | 29.86 ± 0.36 | *F_2,33_* = 0.11; *P* = 0.89 |
| Physical role (SF36) | 20.00 ± 0.00 | 19.10 ± 2.02 | 19.43 ± 1.28 | *F_2,33_* = 1.31; *P* = 0.28 |
| Bodily pain (SF36) | 11.63 ± 0.89 | 12.00 ± 0.00 | 11.43 ± 0.80 | *F_2,33_* = 1.86; *P* = 0.17 |
| General health (SF36) | 23.32 ± 1.77 | 23.24 ± 2.05 | 23.66 ± 1.23 | *F_2,33_* = 0.22; *P* = 0.80 |
| Vitality (SF36) | 15.42 ± 1.31 | 16.50 ± 1.18 | 15.79 ± 1.42 | *F_2,33_* = 1.87; *P* = 0.17 |
| Social function (SF36) | 10.00 ± 0.00 | 9.80 ± 0.63 | 9.36 ± 1.28 | *F_2,33_* = 1.88; *P* = 0.17 |
| Emotional role (SF36) | 14.92 ± 0.29 | 14.10 ± 1.91 | 14.79 ± 0.58 | *F_2,33_* = 1.79; *P* = 0.18 |
| Mental health (SF36) | 21.58 ± 1.44 | 22.30 ± 1.42 | 21.43 ± 2.71 | *F_2,33_* = 0.58; *P* = 0.57 |
| Health transition (SF36) | 2.42 ± 1.08 | 2.50 ± 0.97 | 2.93 ± 0.47 | *F_2,33_* = 1.33; *P* = 0.28 |
| **Eating behaviour (DEBQ)** |  |  |  |  |
| Restrained eating | 2.45 ± 1.17 | 1.86 ± 0.64 | 2.04 ± 0.73 | *F_2,33_* = 1.34; *P* = 0.28 |
| Emotional eating | 1.92 ± 0.64 | 2.15 ± 1.00 | 1.62 ± 0.56 | *F_2,33_* = 1.58; *P* = 0.22 |
| External eating | 3.12 ± 0.58 | 2.98 ± 0.70 | 2.67 ± 0.48 | *F_2,33_* = 2.02; *P* = 0.15 |
| **Intelligence** |  |  |  |  |
| RAVEN | 6.58 ± 1.78 | 5.90 ± 2.42 | 5.64 ± 2.50 | *F_2,33_* = 0.58; *P* = 0.57 |
| Verbal reasoning task | 27.67 ± 10.17 | 18.30 ± 9.59 | 25.36 ± 16.38 | *F_2,33_* = 1.55; *P* = 0.23 |
| **Mood state (PANAS)** |  |  |  |  |
| Positive | 37.17 ± 6.42 | 40.50 ± 3.89 | 38.07 ± 4.41 | *F_2,33_* = 1.25; *P* = 0.30 |
| Negative | 14.33 ± 3.23 | 13.56 ± 3.71 | 14.23 ± 5.13 | *F_2,33_* = 0.10; *P* = 0.90 |
| BFI = Big Five Inventory; BIS/BAS = Behavioural Inhibition System and Behavioural Activation System; SF36 = 36-Item Short-Form Health Survey; DEBQ = Dutch Eating Behaviour Questionnaire; RAVEN = Raven Advanced Progressive Matrices Test; PANAS = Positive and Negative Affect Scale  *For the majority of the participants (n = 30), a one-week actigraphy period was performed two weeks prior to the first laboratory session for the assessment of participants’ habitual sleep-wake schedule, while for the rest (n = 6), since the two laboratory sessions were separated for more than three weeks, habitual sleep-wake timing was re-assessed prior to the second laboratory session as well.  † *p* < .05 for the contrast between *PER3^4/4^* and *PER3^5/5^* individuals | | | | |
