# Supplementary material for: Effects of Partial and Acute Total Sleep Deprivation on Performance across Cognitive Domains, Individuals and Circadian Phase
Source: PLoS One. 2012 Sep 24;7(9):e45987. doi: 10.1371/journal.pone.0045987 (PMC3454374; doi:10.1371/journal.pone.0045987)
Supplement: Table S2 — Results of a general linear mixed model examining the effects of Condition (Sleep Restriction vs. Control) and Day (from baseline to the second day of total sleep deprivation) on performance. (DOC) [file pone.0045987.s012.doc]

**Table S2** Results of a general linear mixed model examining the effects of Condition (Sleep Restriction vs. Control) and Day (from baseline to the second day of total sleep deprivation) on performance

| **Measures** | **Condition** | | | | **Day** | | | | **Condition × Day** | | | |
| --- | --- | --- | --- | --- | --- | --- | --- | --- | --- | --- | --- | --- |
|  | ***F*** | ***df*** | ***f^2^*** | ***p*** | ***F*** | ***df*** | ***f^2^*** | ***p*** | ***F*** | ***df*** | ***f^2^*** | ***p*** |
| **Subjective alertness** |  |  |  |  |  |  |  |  |  |  |  |  |
| KSS | 65.80 | 1,121 | 0.54 | **<0.001** | 91.10 | 9,542 | 1.51 | **<0.001** | 1.64 | 9,542 | 0.03 | 0.10 |
| **Sustained attention** |  |  |  |  |  |  |  |  |  |  |  |  |
| PVT speed | 62.34 | 1,110 | 0.57 | **<0.001** | 92.36 | 9,553 | 1.50 | **<0.001** | 3.13 | 9.553 | 0.05 | **0.001** |
| PVT lapse | 48.54 | 1,125 | 0.39 | **<0.001** | 104.03 | 9,553 | 1.69 | **<0.001** | 3.87 | 9,553 | 0.06 | **<0.001** |
| SART A’ | 47.86 | 1,177 | 0.27 | **<0.001** | 44.86 | 9,550 | 0.73 | **<0.001** | 2.12 | 9,550 | 0.03 | **0.03** |
| **Working memory** |  |  |  |  |  |  |  |  |  |  |  |  |
| V1bk A’ | 19.45 | 1,168 | 0.12 | **<0.001** | 29.01 | 9,554 | 0.47 | **<0.001** | 1.20 | 9,554 | 0.02 | 0.29 |
| V2bk A’ | 32.73 | 1,389 | 0.08 | **<0.001** | 50.02 | 9,606 | 0.74 | **<0.001** | 1.73 | 9,606 | 0.03 | 0.08 |
| V3bk A’ | 14.23 | 1,159 | 0.09 | **0.002** | 29.11 | 9,552 | 0.47 | **<0.001** | 0.74 | 9,552 | 0.01 | 0.67 |
| V1bk bias | 16.35 | 1,147 | 0.11 | **<0.001** | 15.42 | 9,539 | 0.26 | **<0.001** | 1.11 | 9,539 | 0.02 | 0.35 |
| V2bk bias | 27.04 | 1,171 | 0.16 | **<0.001** | 20.39 | 9,519 | 0.35 | **<0.001** | 0.85 | 9,519 | 0.01 | 0.57 |
| V3bk bias | 6.57 | 1,136 | 0.05 | **<0.001** | 7.92 | 9,551 | 0.13 | **<0.001** | 1.99 | 9,551 | 0.03 | **0.04** |
| Note: The model also included the effect of Session (first vs. second visit), but results are not shown. *f^2^* = (*u* / *v*) *F*, where *u* and *v* are respectively the numerator and denominator degrees of freedom of the *F* statistic used to determine the corresponding main or interaction effect in the general linear mixed model analysis. | | | | | | | | | | | | |
