# Supplementary material for: Effects of Partial and Acute Total Sleep Deprivation on Performance across Cognitive Domains, Individuals and Circadian Phase
Source: PLoS One. 2012 Sep 24;7(9):e45987. doi: 10.1371/journal.pone.0045987 (PMC3454374; doi:10.1371/journal.pone.0045987)
Supplement: Table S3 — Effects of repeated partial and acute total sleep deprivation on performance. (DOC) [file pone.0045987.s013.doc]

**Table S3** Effects of repeated partial and acute total sleep deprivation on performance

| **Measures** | **Repeated partial sleep deprivation** | | | | **Acute total sleep deprivation** | | | |
| --- | --- | --- | --- | --- | --- | --- | --- | --- |
|  | ***F*** | ***df*** | ***f^2^*** | ***p*** | ***F*** | ***df*** | ***f^2^*** | ***p*** |
| **Subjective alertness** |  |  |  |  |  |  |  |  |
| KSS | 45.58 | 1,103 | 0.44 | **<0.001** | 212.19 | 1,68.8 | 3.08 | **<0.001** |
| **Sustained attention** |  |  |  |  |  |  |  |  |
| PVT speed | 29.93 | 1,103 | 0.29 | **<0.001** | 210.09 | 1,68.3 | 3.08 | **<0.001** |
| PVT lapse | 21.00 | 1,103 | 0.20 | **<0.001** | 207.77 | 1,68.3 | 3.04 | **<0.001** |
| SART A’ | 19.69 | 1,103 | 0.19 | **<0.001** | 117.63 | 1,68.6 | 1.71 | **<0.001** |
| **Working memory** |  |  |  |  |  |  |  |  |
| V1bk A’ | 12.08 | 1,104 | 0.12 | **<0.001** | 61.93 | 1,68.8 | 0.90 | **<0.001** |
| V2bk A’ | 6.71 | 1,103 | 0.07 | **0.01** | 72.02 | 1,68.8 | 1.05 | **<0.001** |
| V3bk A’ | 4.11 | 1,103 | 0.04 | **0.045** | 29.69 | 1,68.9 | 0.43 | **<0.001** |
| V1bk bias | 5.67 | 1,103 | 0.06 | **0.02** | 54.85 | 1,68.9 | 0.80 | **<0.001** |
| V2bk bias | 11.78 | 1,102 | 0.12 | **<0.001** | 25.72 | 1,69 | 0.37 | **<0.001** |
| V3bk bias | 3.26 | 1,105 | 0.03 | 0.07 | 17.24 | 1,68.6 | 0.25 | **<0.001** |
| Note: The effect of partial sleep deprivation was assessed by comparing performance during D5 and D6 between conditions. The effect of total sleep deprivation was assessed by comparing performance on TD1 to TD2 across conditions. *f^2^* = (*u* / *v*) *F*, where *u* and *v* are respectively the numerator and denominator degrees of freedom of the *F* statistic used to determine the corresponding main effect in the general linear mixed model analysis. | | | | | | | | |
