# Supplementary material for: Effects of Partial and Acute Total Sleep Deprivation on Performance across Cognitive Domains, Individuals and Circadian Phase
Source: PLoS One. 2012 Sep 24;7(9):e45987. doi: 10.1371/journal.pone.0045987 (PMC3454374; doi:10.1371/journal.pone.0045987)
Supplement: Table S4 — Results of a general linear mixed model examining the effects of Genotype, Condition (Sleep Restriction vs. Control), and Day (from baseline to the second day of total sleep deprivation) on performance. (DOC) [file pone.0045987.s014.doc]

**Table S4** Results of a general linear mixed model examining the effects of Genotype, Condition (Sleep Restriction vs. Control), and Day (from baseline to the second day of total sleep deprivation) on performance

| **Measures** | **Genotype** | | | | **Condition** | | | | **Day** | | | | **Genotype × Condition** | | | | **Genotype × Condition × Day** | | | |
| --- | --- | --- | --- | --- | --- | --- | --- | --- | --- | --- | --- | --- | --- | --- | --- | --- | --- | --- | --- | --- |
|  | ***F*** | ***df*** | ***f^2^*** | ***p*** | ***F*** | ***df*** | ***f^2^*** | ***p*** | ***F*** | ***df*** | ***f^2^*** | ***p*** | ***F*** | ***df*** | ***f^2^*** | ***p*** | ***F*** | ***df*** | ***f^2^*** | ***p*** |
| **Subjective alertness** |  |  |  |  |  |  |  |  |  |  |  |  |  |  |  |  |  |  |  |  |
| KSS | 1.00 | 2,33.1 | 0.06 | 0.38 | 64.59 | 1,119 | 0.54 | **<0.001** | 89.84 | 9,509 | 1.59 | **<0.001** | 5.81 | 2,119 | 0.10 | **0.0039** | 0.82 | 18,541 | 0.03 | 0.68 |
| **Sustained attention** |  |  |  |  |  |  |  |  |  |  |  |  |  |  |  |  |  |  |  |  |
| PVT speed | 1.90 | 2,33 | 0.12 | 0.17 | 60.46 | 1,103 | 0.59 | **<0.001** | 90.55 | 9,520 | 1.57 | **<0.001** | 0.28 | 2,103 | 0.0054 | 0.76 | 0.49 | 18,548 | 0.02 | 0.96 |
| PVT lapse | 1.82 | 2,33.1 | 0.11 | 0.18 | 46.47 | 1,118 | 0.39 | **<0.001** | 101.91 | 9,520 | 1.76 | **<0.001** | 0.37 | 2,118 | 0.0063 | 0.69 | 0.48 | 18,548 | 0.02 | 0.97 |
| SART A’ | 0.19 | 2,33.1 | 0.01 | 0.82 | 49.64 | 1,166 | 0.30 | **<0.001** | 43.75 | 9,517 | 0.76 | **<0.001** | 2.46 | 2,166 | 0.03 | 0.09 | 0.52 | 18,547 | 0.02 | 0.95 |
| **Working memory** |  |  |  |  |  |  |  |  |  |  |  |  |  |  |  |  |  |  |  |  |
| V1bk A’ | 3.03 | 2,33.2 | 0.18 | 0.06 | 18.68 | 1,154 | 0.12 | **<0.001** | 27.40 | 9,522 | 0.47 | **<0.001** | 0.46 | 2,154 | 0.0060 | 0.63 | 0.61 | 18,550 | 0.02 | 0.90 |
| V2bk A’ | 1.33 | 2,33 | 0.08 | 0.28 | 32.97 | 1,370 | 0.09 | **<0.001** | 48.12 | 9,572 | 0.76 | **<0.001** | 0.71 | 2,370 | 0.0038 | 0.49 | 0.53 | 18,583 | 0.02 | 0.94 |
| V3bk A’ | 1.20 | 2,33.1 | 0.07 | 0.31 | 14.07 | 1,155 | 0.09 | **<0.001** | 28.94 | 9,520 | 0.50 | **<0.001** | 3.42 | 2,155 | 0.04 | **0.04** | 0.81 | 18,549 | 0.03 | 0.69 |
| V1bk bias | 1.29 | 2,32.7 | 0.08 | 0.29 | 14.03 | 1,136 | 0.10 | **<0.001** | 14.53 | 9,509 | 0.26 | **<0.001** | 0.99 | 2,136 | 0.01 | 0.37 | 0.76 | 18,541 | 0.03 | 0.75 |
| V2bk bias | 1.04 | 2,32.6 | 0.06 | 0.37 | 27.01 | 1,157 | 0.17 | **<0.001** | 20.52 | 9,481 | 0.38 | **<0.001** | 1.06 | 2,156 | 0.01 | 0.35 | 1.13 | 18,523 | 0.04 | 0.32 |
| V3bk bias | 0.31 | 2,33.3 | 0.02 | 0.74 | 6.13 | 1,127 | 0.05 | **0.01** | 7.83 | 9,518 | 0.14 | **<0.001** | 1.52 | 2,127 | 0.02 | 0.22 | 1.13 | 18,547 | 0.04 | 0.31 |
| Note: The model also included the effect of Session (first vs. second visit), but results are not shown. *f^2^* = (*u* / *v*) *F*, where *u* and *v* are respectively the numerator and denominator degrees of freedom of the *F* statistic used to determine the corresponding main or interaction effect in the general linear mixed model analysis. | | | | | | | | | | | | | | | | | | | | |
