# Supplementary material for: Effects of Partial and Acute Total Sleep Deprivation on Performance across Cognitive Domains, Individuals and Circadian Phase
Source: PLoS One. 2012 Sep 24;7(9):e45987. doi: 10.1371/journal.pone.0045987 (PMC3454374; doi:10.1371/journal.pone.0045987)
Supplement: Table S5 — Circadian phase markers of the PER34/4 , PER34/5 , and PER35/5 participants. (DOC) [file pone.0045987.s015.doc]

**Table S5** Circadian phase markers of the *PER3^4/4^*, *PER3^4/5^*, and *PER3^5/5^* participants

| **Circadian phase markers** |  | **Mean ± standard error** | | | ***P*** | | | |
| --- | --- | --- | --- | --- | --- | --- | --- | --- |
|  | **Cond** | ***PER3^4/4^*** | ***PER3^4/5^*** | ***PER3^5/5^*** | **Gene** | **Cond** | **Session** | **Gene×Cond** |
| **Dim light melatonin** |  |  |  |  |  |  |  |  |
| Onset (25%) | C | 23:58 ± 00:27 | 23:30 ± 00:27 | 22:55 ± 00:24 | 0.25 | **< 0.0001** | 0.41 | 0.43 |
|  | SR | 00:46 ± 00:27 | 00:00 ± 00:26 | 23:52 ± 00:24 |  |  |  |  |
| Mid-point (25%) | C | 04:49 ± 00:25 | 04:29 ± 00:26 | 03:46 ± 00:23 | 0.21 | **0.0005** | 0.34 | 0.09 |
|  | SR | 05:29 ± 00:25 | 04:34 ± 00:25 | 04:34 ± 00:23 |  |  |  |  |
| Offset (25%) | C | 09:40 ± 00:26 | 09:22 ± 00:27 | 08:37 ± 00:24 | 0.21 | 0.05 | 0.50 | 0.06 |
|  | SR | 10:13 ± 00:26 | 09:07 ± 00:26 | 09:16 ± 00:24 |  |  |  |  |
| Onset (50%) | C | 00:45 ± 00:28 | 00:20 ± 00:28 | 23:42 ± 00:25 | 0.32 | **< 0.0001** | 0.84 | 0.10 |
|  | SR | 01:28 ± 00:28 | 00:38 ± 00:27 | 00:41 ± 00:25 |  |  |  |  |
| Mid-point (50%) | C | 04:45 ± 00:25 | 04:13 ± 00:25 | 03:36 ± 00:23 | 0.16 | **0.0001** | 0.90 | 0.12 |
|  | SR | 05:23 ± 00:25 | 04:25 ± 00:25 | 04:25 ± 00:23 |  |  |  |  |
| Offset (50%) | C | 08:45 ± 00:25 | 08:06 ± 00:25 | 07:29 ± 00:23 | 0.08 | **0.0082** | 0.99 | 0.31 |
|  | SR | 09:18 ± 00:25 | 08:12 ± 00:25 | 08:08 ± 08:23 |  |  |  |  |
| **Fitted curve** |  |  |  |  |  |  |  |  |
| Time of peak | C | 04:51 ± 00:26 | 04:21 ± 00:26 | 03:47 ± 00:23 | 0.20 | **0.0014** | 0.81 | 0.16 |
|  | SR | 05:25 ± 00:26 | 04:27 ± 00:26 | 04:30 ± 00:23 |  |  |  |  |
| Amplitude | C | 40.39 ± 8.46 | 39.38 ± 8.46 | 55.40 ± 7.72 | 0.30 | 0.58 | 0.99 | 0.90 |
|  | SR | 39.76 ± 8.46 | 39.41 ± 8.45 | 54.37 ± 7.72 |  |  |  |  |
| Means and standard errors were the least squares means and standard errors estimated by PROC MIXED in SAS.  Cond = condition; Gene = genotype; Session (first or second visit); C = Control condition; SR = Sleep Restriction condition.  Percentage in brackets indicates the level of melatonin from which the corresponding circadian phase marker was derived; see the Materials and Methods section for details. | | | | | | | | |
