# Supplementary material for: Effects of Partial and Acute Total Sleep Deprivation on Performance across Cognitive Domains, Individuals and Circadian Phase
Source: PLoS One. 2012 Sep 24;7(9):e45987. doi: 10.1371/journal.pone.0045987 (PMC3454374; doi:10.1371/journal.pone.0045987)
Supplement: Table S6 — Results of a general linear mixed model examining the effects of Condition (Sleep Restriction vs. Control) and Circadian Phase (between DLMO 12 h to DLMO+20 h) on performance. (DOC) [file pone.0045987.s016.doc]

**Table S6** Results of a general linear mixed model examining the effects of Condition (Sleep Restriction vs. Control) and Circadian Phase (between DLMO ~~-~~ 12 h to DLMO + 20 h) on performance

| **Measures** | **Condition** | | | | **Circadian Phase** | | | | **Condition**  **× Circadian Phase** | | | |
| --- | --- | --- | --- | --- | --- | --- | --- | --- | --- | --- | --- | --- |
|  | ***F*** | ***df*** | ***f^2^*** | ***p*** | ***F*** | ***Df*** | ***f^2^*** | ***p*** | ***F*** | ***df*** | ***f^2^*** | ***p*** |
| **Subjective alertness** |  |  |  |  |  |  |  |  |  |  |  |  |
| KSS | 28.50 | 1,88.6 | 0.32 | **<0.001** | 49.01 | 8,493 | 0.80 | **<0.001** | 2.33 | 8,493 | 0.04 | **0.02** |
| **Sustained attention** |  |  |  |  |  |  |  |  |  |  |  |  |
| PVT speed | 49.38 | 1,91.4 | 0.54 | **<0.001** | 52.01 | 8,493 | 0.84 | **<0.001** | 2.53 | 8,493 | 0.04 | **0.01** |
| PVT lapse | 31.16 | 1,98.7 | 0.32 | **<0.001** | 49.26 | 8,496 | 0.79 | **<0.001** | 3.93 | 8,496 | 0.06 | **<0.001** |
| SART A’ | 46.00 | 1,156 | 0.29 | **<0.001** | 27.90 | 8,486 | 0.46 | **<0.001** | 1.37 | 8,486 | 0.02 | 0.21 |
| **Working memory** |  |  |  |  |  |  |  |  |  |  |  |  |
| V1bk A’ | 18.07 | 1,155 | 0.12 | **<0.001** | 17.52 | 8,488 | 0.29 | **<0.001** | 0.54 | 8,488 | 0.01 | 0.83 |
| V2bk A’ | 21.08 | 1,143 | 0.15 | **<0.001** | 18.91 | 8,490 | 0.31 | **<0.001** | 1.14 | 8,490 | 0.02 | 0.33 |
| V3bk A’ | 11.83 | 1,136 | 0.09 | **<0.001** | 13.64 | 8,491 | 0.22 | **<0.001** | 0.56 | 8,491 | 0.01 | 0.81 |
| V1bk bias | 15.03 | 1,135 | 0.11 | **<0.001** | 13.07 | 8,480 | 0.22 | **<0.001** | 1.05 | 8,480 | 0.02 | 0.40 |
| V2bk bias | 12.00 | 1,154 | 0.08 | **<0.001** | 10.59 | 8,474 | 0.18 | **<0.001** | 0.96 | 8,474 | 0.02 | 0.47 |
| V3bk bias | 5.26 | 1,121 | 0.04 | **0.02** | 6.08 | 8,477 | 0.10 | **<0.001** | 0.79 | 8,477 | 0.01 | 0.61 |
| Note: The model also included the effect of Session (first vs. second visit), but results are not shown. *f^2^* = (*u* / *v*) *F*, where *u* and *v* are respectively the numerator and denominator degrees of freedom of the *F* statistic used to determine the corresponding main or interaction effect in the general linear mixed model analysis. | | | | | | | | | | | | |
