# Supplementary material for: Effects of Partial and Acute Total Sleep Deprivation on Performance across Cognitive Domains, Individuals and Circadian Phase
Source: PLoS One. 2012 Sep 24;7(9):e45987. doi: 10.1371/journal.pone.0045987 (PMC3454374; doi:10.1371/journal.pone.0045987)
Supplement: Table S7 — The three orders of the cognitive tasks included in the test battery. (DOC) [file pone.0045987.s017.doc]

**Table S7** The three orders of the cognitive tasks included in the test battery

| **Task number** | **Order 1** | **Order 2** | **Order 3** |
| --- | --- | --- | --- |
| 1 | Karolinska Drowsiness Test (KDT) | KDT | KDT |
| 2 | Karolinska Sleepiness Scale (KSS) | KSS | KSS |
| 3 | Pursuit Tracking Task (PTT) | S1- and S2-back | PIR, RIR |
| 4 | Positive And Negative Affect Scale (PANAS) | P1- and P2-back | PVT |
| 5 | Spatial 1- and 2-back (S1- and S2-back) | I1- and I2-back | SART |
| 6 | Pictorial 1- and 2-back (P1- and P2-back) | PVT | PTT |
| 7 | Integrated 1- and 2-back (I1- and I2-back) | SART | PANAS |
| 8 | Psychomotor Vigilance Task (PVT) | PIR, RIR | V1-Back, VAS, V2-Back, VAS, V3-Back, and VAS |
| 9 | Sustained Attention Response Task (SART) | V1-Back, VAS, V2-Back, VAS, V3-Back, and VAS | P1- and P2-back |
| 10 | Verbal 1-, 2-, and 3-back (V1-, V2-, and V3-back) each followed by a Visual Analogue Scale (VAS) | PTT | S1- and S2-back |
| 11 | Fixed Interval Repetition (FIR) and Random Interval Repetition (RIR) | PANAS | I1- and I2-back |
| 12 | KDT | KDT | KDT |
| 13 | KSS | KSS | KSS |
